# Self-organization of self-clearing beating patterns in an array of locally interacting ciliated cells formulated as an adaptive Boolean network

**DOI:** 10.1007/s12064-019-00299-x

**Published:** 2019-07-26

**Authors:** Martin Schneiter, Jaroslav Rička, Martin Frenz

**Affiliations:** Institute of Applied Physics, University of Bern, Sidlerstrasse 5, 3012 Bern, Switzerland

**Keywords:** Cilia, Unciliated cells, Self-organized mucociliary clearance, Synchronization, Modularity, Adaptivity, Structure emergence

## Abstract

The observed spatiotemporal ciliary beat patterns leading to proper mucociliary clearance on multiciliated epithelia are suspected to be the result of self-organizing processes on various levels. Here, we present a simplified pluricellular epithelium model, which intends to make the self-organization of ciliary beating patterns as well as of the associated fluid transport across the airway epithelium plausible. The model is based on a two-dimensional array of locally interacting oscillating ciliated cells. Ciliated cells are represented by Boolean actuators, and abstracted hydrodynamic mucociliary interactions are formulated in terms of logical update rules (Boolean functions). In the course of a simulation, initial random conformations of an array of actuators self-organize toward metachronally coordinated states exhibiting efficient transport of mucus. Within the framework of Boolean networks ciliated cells represent the nodes of the network and as the mucus establishes the local interactions among nodes, its distribution (together with the formulated local interactions) determines the topology of the network. Consequently, we propose to consider the dynamics on multiciliated epithelia in the context of adaptive (Boolean) networks. Furthermore, we would like to present insights gained from conducted comprehensive parameter studies. In particular, the dynamical response of the network with respect to variations of the boundary conditions, updating schemes (representing intercellular signaling mechanisms) and the proportion of ciliated cells is presented.

**Electronic supplementary material:**

The online version of this article (10.1007/s12064-019-00299-x) contains supplementary material, which is available to authorized users.

## Introduction

Motivation for the present work is to contribute to the understanding of the fascinating and omnipresent phenomenon of mucociliary transport.

The epithelium of our airways constitutes a self-cleaning surface protecting our lungs from a variety of inhaled substances such as exhaust, dust, bacteria and other harmful substances of micro- and submicrometer size. These particles get entrapped by the mucus layer lining the inner surface of the tracheal and pulmonary airways, which is propelled by the coordinated oscillatory movement of millions of subjacent cilia. Cilia are hair-like protrusions of the cell membrane; an overview of their structure and function has been provided by Linck (2009) and Satir and Christensen (2007).

To gain insight into the mucociliary clearing mechanism, many experimental techniques on various length- and time-scales are conducted. Experimental studies typically focus either on a structural or functional component on a typical scale, like the remarkable insight into the detailed molecular structure of the axoneme (see, e.g., Burgess et al. 2003; Sui and Downing 2006), the orientation of the ciliary beating plane (Satir and Christensen 2007), the distribution of the different epithelial cell types (e.g., Plopper et al. 1983; Oliveira et al. 2003) or the observation of mucociliary phenomena on the pluricellular level (Ryser et al. 2007). Even though much work has been done on various scales, our understanding of the basic mesoscopic function of the system still appears rather limited. Experimental methods face several challenges: until today, it is not possible to observe the details of the mucociliary dynamics in vivo. Further, it is difficult to observe the mucociliary clearing mechanism under controlled conditions. And finally, it is highly complex and laborious to simultaneously measure structural and functional parameters at different scales.

Mathematical models are perfectly suited to conduct parameter studies in order to determine the effects of structural and functional parameters on mucociliary phenomena and therefore, serve as an alternative approach for the investigation of the intriguing mucociliary phenomena. Considering the implementation of mucociliary interactions, the existing models can roughly be divided into two classes.

One class of models prescribes the motion of cilia, including their coordination, and concentrates on the hydrodynamics and rheology of the system. In these models, the action of cilia is modeled as distributed oscillating momentum source, such as oscillating envelope (Ross and Corrsin 1974), traction layer (Smith et al. 2007a), active porous medium or oscillating array of cilia represented by a distribution of hydrodynamic singularities along the cilia’s centerline (Smith et al. 2007b). The aim of these studies is to elaborate the geometrical and rheological conditions under which the system achieves an efficient transport (e.g., Lee et al. 2011).

More relevant to the context of the present study is a second class of models, aimed at the understanding of the emergence of the pattern of motion. In these models, the details of the motion of cilia or their coordination are not prescribed explicitly. The coordinated behavior rather emerges in the course of self-organization, during which the system components interact locally, what drives the system from an initially uncoordinated state toward a globally coordinated state exhibiting cooperative behavior.

Self-organization may play a role on various time- and length-scales, at various levels for the generation of mucociliary transport. As discussed by Marshall (2010), the interactions between cilia-generated fluid flow and planar cell polarity (PCP) signaling may lead to self-organization during the morphogenesis, which establishes the alignment of the axonemes on the individual cells. Much of work has been done on the molecular level, concerning the molecular motors (dynein motor proteins), their organization in the axoneme as well as the hydrodynamics of the resulting model cilium (Riedel-Kruse et al. 2007; Hilfinger and Jülicher 2008; Hilfinger et al. 2009). The complex motion pattern of a single cilium is thought to result from the self-organization of many dynein motor proteins generating stresses on the elastic microtubules in the axoneme. The next higher level, the cellular level, concerns the formation of metachronal waves by the self-organized synchronization of cilia covering a single cell (Elgeti and Gompper 2013). For a long time, it remained an open question whether the synchronization is achieved by cellular signaling (membrane potentials and calcium waves), or if it emerges spontaneously, due to interactions between the individual cilia. Today, the computational models indicate that hydrodynamic coupling is sufficient to induce synchronization (Elgeti and Gompper 2013; Mitran 2007; Gueron et al. 1997). It is conceivable, however, that calcium signaling is needed for fine tuning the synchronization (Salathe 2007).

Here, we propose a first step to the next higher level, the pluricellular level, on which the self-organization of ciliary activity among ciliated cells is thought to generate the global wave field and fluid transport on the airway epithelium. In order to make the self-organized beat patterns as well as self-organized fluid transport across the airway epithelium plausible, we present a simplified model, which is intended to represent a virtual self-cleaning epithelium. Ciliated cells are modeled as actuators alternating between two possible states representing ciliary oscillations. Interactions between the two-state actuators are mediated by discrete mucus droplets and enmeshed dust particles. Whenever it is possible, the mucus droplets get displaced by the action of actuators and they may block their motion in certain configurations, which is prescribed by local interaction rules. This highly simplified model based on locally interacting motors self-organizes toward a virtual self-cleaning epithelium: the initially randomly distributed phases erratically displace the mucus lumps at the beginning of the simulation. As time passes the motors self-organize, which is expressed by emergent global spatiotemporal structures, resembling the metachronal wavelets, which have been observed on the ciliated tracheal epithelium (Ryser et al. 2007). These metachronal wavelets efficiently transport the mucus lumps into a well-defined direction.

This paper is organized as follows. In “Model description” section, we introduce our abstract epithelium model. First, an introduction and biophysical motivation of the various model components, such as different morphologies, boundary conditions and update schemes, is provided. In the last section of the model description (“Concise formal model description” section ), we provide a formal complete description of the model. Therefore, readers with a strong mathematical background principally have the possibility to skip the “Symmetrically interacting two-state actuators” section –“Boolean network representation” section. The goal of “Model description” section is to formulate our epithelium model in terms of an adaptive Boolean network. Within the framework of adaptive Boolean networks, nodes are represented by actuators and the topology of the network (in effect the links between the nodes) is determined by the mucus distribution. Thanks to the simplicity of our epithelium model, it is possible to investigate the impact of the model parameters on the system’s behavior by conducting comprehensive parameter studies. The corresponding simulation data are presented in “Simulations and results” section. The simulation data were screened for any suspicious coherences between the model parameters and the network dynamics as well as between the parameters and their corresponding attracting states. Furthermore, we aimed at finding the mechanisms driving the model efficiently toward properly self-cleaning states. Finally, in “Discussion” section we discuss our findings. In particular, we hypothesize about the meaning of the discovered dynamical aspects, which seem to be universal for our specific model of locally interacting cells, for the real biological system.

## Model description

### Symmetrically interacting two-state actuators

**Fig. 1:**
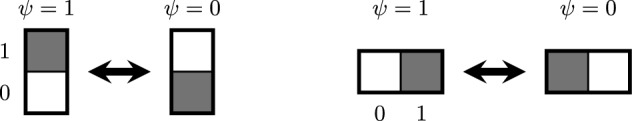
Ciliated cells are represented by two-state actuators. An actuator’s current state is expressed by the Boolean state variable $\psi$. Each actuator provides two fields (0 and 1)

Our model is based on symmetrically interacting two-state actuators. Each actuator represents a ciliated epithelial cell. By arranging many actuators in a parquet-like manner, the model represents a ciliated epithelium. Cilia corresponding to the same cell are assumed to move synchronously back and forth. This back and forth motion of cilia bundles is incorporated by the alternation between the actuators’ two possible states, which is illustrated in Fig. 1. The actual state of an actuator can thus be expressed by the Boolean state variable $\psi \in \{0,1\}$.

### Morphology of virtual epithelia

The morphology of the airway epithelium is thought to be the result of self-organizing processes during the morphogenesis (Marshall 2010). An important characteristic of the morphology is the distribution of the orientation of the ciliary beating plane, which can be determined by the orientation of the microtubules in the axoneme (Satir and Christensen 2007). The impact of the (dis-)orientation of the ciliary beating plane, i.e., of the axonemal orientation on mucociliary transport, has been discussed, e.g., by Rutland and De Iongh (1993) and De Iongh and Rutland (1989). It has been concluded that a disorganized ciliary orientation may be a primary cause for mucociliary dysfunction and vice versa. Studies attempting to quantify the axonemal orientation of respiratory cilia have usually considered the ciliary beating plane on a few cells derived from nasal brushing (e.g., Rautiainen 1988). Here, we first of all assume that cilia belonging to the same cell have a coinciding beating plane. As it is conceivable that the ciliary orientation on a cell might be more strict than between cells, the actuators can be oriented vertically or horizontally, as illustrated in Fig. 1. Note that the two different possible orientations are not meant being perpendicular to each other, but to represent two distinct orientations of the ciliary beating plane differing by an arbitrary angle between cells.

**Fig. 2:**
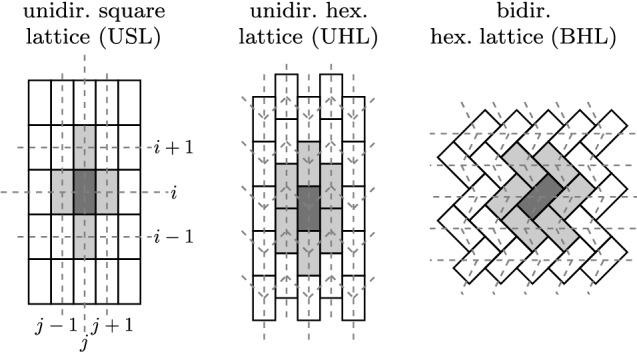
Three different cell alignments have been investigated. Bright gray colored cells label the neighborhood of a central cell colored in dark gray. Dashed grid lines indicate the mapping onto a two-dimensional array

Here, we consider three different conceivable parquet-like cell alignments shown in Fig. 2. In the following, we shall refer to these three cell alignments as unidirected square lattice (USL), unidirected hexagonal lattice (UHL) and bidirected hexagonal lattice (BHL).

Another important characteristic of the morphology represents the population densities of ciliated and unciliated cells, whose role for the mucociliary dynamics has not been considered so far. According to electron microscopic studies (Plopper et al. 1983; Oliveira et al. 2003), the area covered by ciliated cells roughly varies between one and two-thirds of the total surface of the tracheal lining. Consequently, unciliated cells should be considered in pluricellular models. Therefore, we included the proportion $f \in [0,1]$ of unciliated epithelial cells, which are represented by randomly distributed empty sites in an array of actuators.

Finally, as indicated by the dashed grid lines in Fig. 2 the virtual epithelium is represented by a two-dimensional array. For convenience, a site located at the *i*-th row and *j*-th column is denoted as $\psi _{ij}$. $\psi _{ij}$ either represents an actuator, then $\psi _{ij} \in \{0,1\}$, or indicates an empty site representing an unciliated cell, then $\psi _{ij}=\hbox {NaN}$. Accordingly, the state of an array of cells with *I* rows and *J* columns can be denoted as: $\varPsi = \{\psi _{ij}\}$, where $\psi _{ij} \in \{0,1,\text {NaN}\}$.

### Boundary conditions

**Fig. 3:**
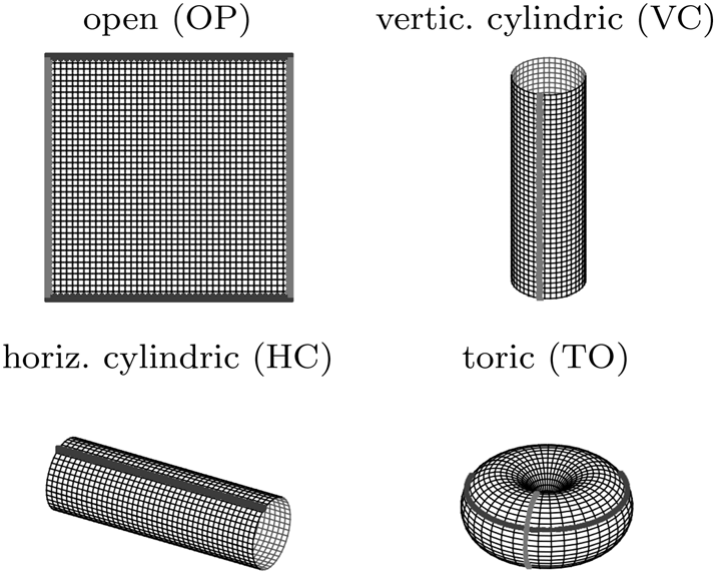
Boundary conditions: open, vertical cylindrical, horizontal cylindrical and toric boundaries. The dark gray and bright gray lines indicate wrapped boundaries

Experiments aiming at the characterization of spatiotemporal features of mucociliary phenomena on the tracheal ciliary epithelium are based on the approach of excising a rectangular piece of the cylindric trachea (Wong et al. 1993; Yi et al. 2002; Lee et al. 2005; Ryser et al. 2007), which changes the boundary conditions from cylindrical to open. The excision might influence the collective dynamical behavior of the system. Therefore, we consider four different boundary conditions, which are illustrated in Fig. 3 and shall be referenced in the following as: open boundaries (OP), vertical cylindric boundaries (VC), horizontal cylindric boundaries (HC) and toric boundaries (TO).

### Mucus

Mucus is discontinuously incorporated by randomly seeding mucus droplets of equal size. On the one hand, the discrete mucus model allows to simplify mucociliary interactions. On the other hand, it accounts for the fact that the mucus blanket is anyway made up of excreted mucus “flakes,” “plaques” or ”droplets“ (Van As and Webster 1974), which may coalesce into a continuous layer, if their density is sufficiently large (Geiser et al. 1997).

According to Fig. 1, each actuator provides an empty field (0 or 1), which can be occupied by mucus droplets. On the other hand, empty sites (unciliated cells) provide two fields (0 and 1), which can be occupied by mucus droplets. Thus, the distribution of mucus droplets on the virtual epithelium is given by $\mathbf{M } = \{m_{ijk}\}$, with $k \in \{0,1\}$ specifying the field within a site *ij*. In order to simplify the notation, the term $m_{ij}$ refers to $\sum _{k} m_{ijk}$ in the following.

### System update

**Fig. 4:**
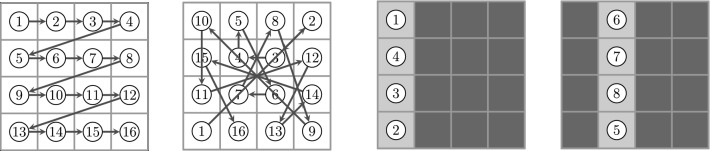
The figure illustrates different update schemes for a lattice consisting of $4 \times 4$ cells. The numbers indicate the order of activation (for one certain time step). From left to right: a pre-defined sequence (DAU), random cell selection without replacement (RAU1) and a planar wavelike activation (SRAU1)

**Fig. 5:**
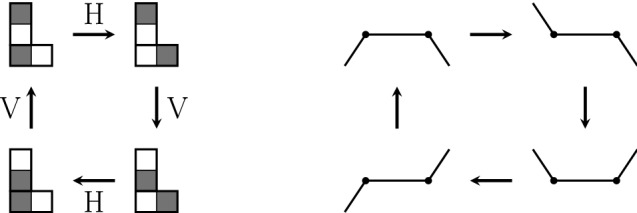
The prescribed cyclic four-phase motion of two adjacent actuators forming the shape of an “L” (left). The construction of this four-phase motion-sequence was motivated by Purcell’s two-hinged swimmer (right)

In the course of a simulation, the actuators are actuated sequentially. This means that only the state of the actuated actuator and its adjacent mucus configuration is updated, while the states of adjacent actuators do not change. As soon as an actuator and its local mucus configuration has been updated, a subsequent actuator is updated for which the changes of the prior step are taken into account. We shall label the sequence of update steps by the “time” superscript *t*. Furthermore, $t^{\prime } \doteq t / N$ labels the update of the whole network, consisting of *N* actuators, in the following. In the context of discrete dynamical systems such sequential update schemes are called asynchronous. An excellent overview of the impact of different update schemes on the network dynamics of random Boolean networks has been provided by C. Gershenson (Gershenson 2002, 2004b). In order to distinguish the different update schemes, we shall use a slightly modified form of the terms proposed in Gershenson (2002) (since we are not dealing with random Boolean networks). We shall use the following update schemes (consider the corresponding illustrations in Fig. 4):

- *DAU—deterministic asynchronous update*
  This update mode corresponds to a pre-defined sequence, in which the nodes/cells in the lattice are addressed. Keep in mind, however, that determinism of addressing does not mean determinism of actuators motion. Whether an actuator moves or not depends on the mucociliary interactions, which are on a high degree stochastic, as we shall see in the proceeding sections.
- *RAU—random asynchronous update*
  At each time step, a single node is chosen randomly and updated. The random choice of nodes has been applied for sampling with and without replacement (Cornforth et al. 2005). Random sequences of nodes generated without and with replacement are denoted as RAU1 and RAU2, respectively.
- *SRAU—semi-random asynchronous update*
  The update scheme SRAU uses a wavelike activation: at each time step a strip of actuators gets addressed. Inside of each strip the actuators get addressed according to the RAU1 scheme. As the choice of the strip is pre-defined but inside the strips the sequence of actuators is chosen randomly, this addressing scheme has a semi-random character. This wavelike activation has been implemented for a wave traveling from the left to the right, as well as for a wave traveling from the top to the bottom, which we denote as SRAU1 and SRAU2, respectively.
- *Mimicking Purcell’s two-hinged low-Reynolds-number-swimmer*
  As non-reciprocal motions play an important role in a low-Reynolds-number environment the cell arrangement BHL has been used together with a prescribed addressing sequence of two neighboring actuators, V and H, forming the shape of an “L.” The two actuators are addressed in the sequence VHVHVH, which would result in a four-phase cyclic motion if the actuators were to move unhinderedly. This scheme, originally proposed by one of the authors in an essay in a popular scientific journal (Ricka 2010), was motivated by the “two-hinged low-Reynolds-number-swimmer,” which has been introduced by Purcell (1977) and is illustrated in Fig. 5. The prescribed addressing sequence principally represents a special locally deterministic update scheme, as it considers the actual state of two locally coupled cells to determine which actuator will be addressed. The selection of the “L” is based on the update schemes introduced above (RAU1, RAU2, SRAU1, SRAU2 and DAU). Consequently, we actually mixed a local update scheme (prescribing the four-phase sequence of an “L”) with different global update schemes (selecting which “L” to address). Settings using the coupling of two cells forming an “L,” will be accounted for a cell arrangement, referred to “BHL + L.”

The different update schemes have been applied in order to investigate the role of potential intercellular signaling mechanisms on the airway epithelium for the dynamical behavior of locally interacting ciliated cells.

### Local mucociliary interactions

For the sake of simplicity, we shall assume from here on that the cells are aligned according to the square-lattice alignment (USL, Fig. 2).

**Fig. 6:**
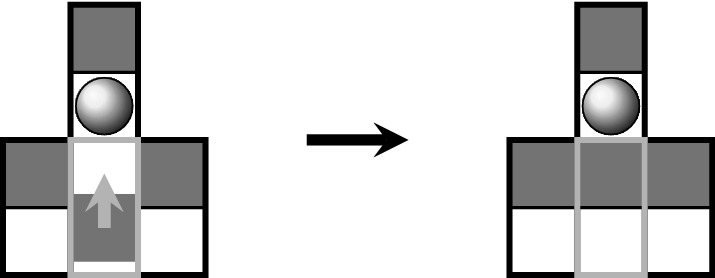
An activated mucus-free actuator (indicated by the bright surround) is always able to alternate its state ($\psi _{ij}^{t+1} = \overline{\psi _{ij}^t}$)

**Fig. 7:**
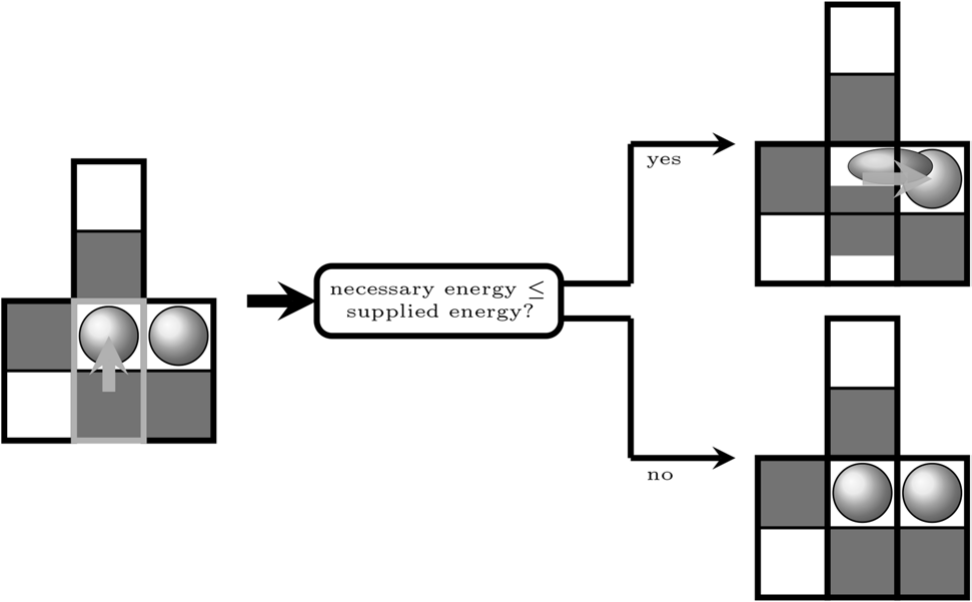
If the active actuator holds a mucus droplet ($m_{ij}^t > 0$), the actuator is either going to stagnate (i.e., $u_{ij}^t < \Delta w$), or the previously ascribed actuator energy is sufficient to switch its state by squeezing the mucus droplets on adjacent fields ($u_{ij}^t \ge \Delta w$)

**Fig. 8:**
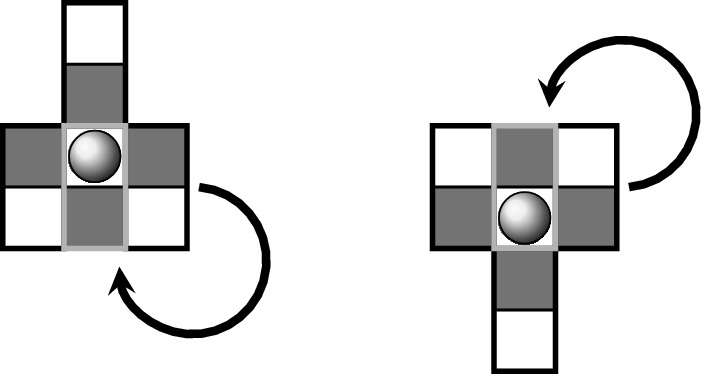
In certain state configurations, the active actuator is locked and consequently remains in its actual state: $\psi _{ij}^{t+1} = \psi _{ij}^t$

Hydrodynamic interactions between adjacent ciliated cells are considered in a simplified fashion and implemented in terms of logical local decision rules induced by mucus droplets randomly seeded on the empty fields of the network. The system’s evolution is achieved by attempting to sequentially move the individual actuators. As interactions between actuators only occur if mucus is located on the activated actuator *ij*, an activated actuator can switch its state unhinderedly as long as there is no mucus, i.e., $m_{ij}^t=0$, hindering its oscillation (Fig. 6). There are, however, two possibilities that an activated actuator *ij*, with a mucus load of $m_{ij}^t > 0$, remains in its actual state. Either the active actuator has not enough energy to shift or squeeze the mucus droplets on adjacent fields (Fig. 7), or the actuator is situated in a locked configuration (see Fig. 8).

Note that there are always two possible locked configurations for each actuator. If the activated actuator *ij* is not situated in a locked configuration, and $m_{ij}^t > 0$, it either flips its state by squeezing the concerned mucus on adjacent fields, or it stagnates and remains in its current state (Fig. 7). Prior to its attempt to move, each actuator *ij* gets a certain amount of inversely sampled energy $u_{ij}^t$ ascribed. If the actuator’s ascribed energy exceeds the required moving energy $\Delta w_{ij}^t$, the actuator is able to move. This way, the actuators’ energy distribution and the situation-specific change in free energy $(\Delta w_{ij}^t)$ determines the probability for an actuator to flip its state by squeezing the mucus on adjacent actuators.

Intercellular coordination, or rather the emergence of global spatiotemporal patterns, are caused by stagnating actuators as well as locked configurations, as in these situations the activated actuator has to adjust its state according to its locally surrounding state and mucus distribution.

### Actuator energy and mucus relaxation

**Fig. 9:**
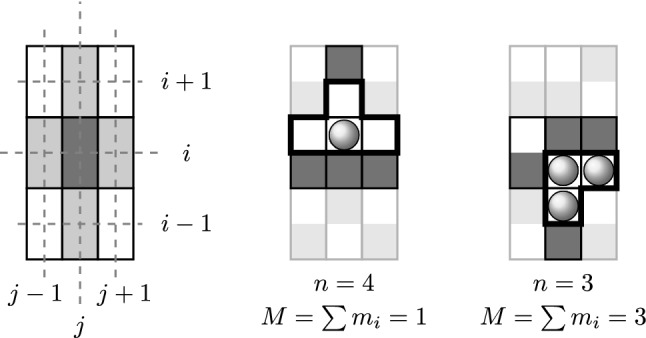
The left panel illustrates the neighborhood of actuators aligned in the square lattice. Bright gray actuators control the subsequent state of the actuated dark gray actuator. Middle and right panel: two situations which are thought to illustrate the locally concerned volume $V=n\Delta V$ and the involved number of mucus droplets *M*. As indicated by the black surround the update of $\psi _{ij}$, as well as of the associated local mucus distribution, depends on a maximum of four sites $(n\le 4)$. The state and mucus distribution of actuators, which are not involved to the current interaction, are irrelevant for the update of $m_{ij}^t$ and $\psi _{ij}^t$. Therefore, these actuators are shown grayed out.

The interactions are quantified using a simple scheme intended to model in a crude fashion the transient entropic elasticity and relaxation of entangled mucin chains. We view the actuator as a piston acting against the pressure *p* exerted by mucus droplets contained in a certain volume *V*. We assume that the interactions are mediated only through *n* adjacent fields of the targeted site (Fig. 9). As a result the volume to be compressed by the action of the piston is $V = n \Delta V$. By moving the actuator, this volume changes by $-\Delta V$ to $V' = (n-1) \Delta V$, what requires the work $\Delta w = p \Delta V$. To determine the pressure *p* we employ the standard thermodynamic relation: $p / T = \partial S(V) / \partial V$, where $S(V) = k_B\text {ln}(W_V)$ is the Boltzmann entropy of the mucus droplets enclosed within *V*.

Replacing the differentials by discrete differences $\partial S \rightarrow \Delta S = k_B\text {ln}(W_{V'}/W_V)$ and $\partial V \rightarrow -\Delta V$ yields

1 $$\begin{aligned} \Delta w = p\Delta V = k_B T \text {ln}\big (\frac{W_n}{W_{n-1}}\big ). \end{aligned}$$

Subsequently, we set $k_BT = 1$, i.e., energy is measured in units of $k_BT$. A plausible expression for the multiplicity (“thermodynamic probability”) $W_n$ can be deduced as follows: random deposition of *M* mucus droplets on *n* available fields is equivalent to rolling an *n*-sided dice. Thus, the result of the deposition of *M* droplets is a sample from the multinomial distribution, with equal probabilities for hitting a field unoccupied by an actuator. Thus, the multinomial coefficient $C_n = M!/(m_1!m_2! \cdot \cdot \cdot m_n!)$ is a good candidate for the multiplicity in Boltzmann entropy. ($m_i$ denotes the number of droplets deposited on a field *i*.) However, after the deposition, prior to an attempted move of an actuator, we allow the distribution of droplets to relax to a “thermodynamic equilibrium,” i.e., into a state of maximum multiplicity, where the droplets are most uniformly distributed on the available fields, so that $|m_i - m_j| \le 1$, for $i \ne j$. In other words, we invoke the maximum entropy principle (Hanel et al. 2014). Therefore, we define the multiplicity involved in Eq. 1 as $W_n=\text {max}\big (M!/m_1!m_2!\cdot \cdot \cdot m_n!\big )$ and $W_{n-1} = \text {max}\big (M!/m_1'!m_2'!\cdot \cdot \cdot m_{n-1}'!\big )$, where $m_i$ and $m_i'$ are subject to the constraints $\sum _{i=1}^{n} m_i = \sum _{i=1}^{n-1} m_i'= M$.

To complete the specification of the mucociliary interactions, we must specify the actuators’ ascribed energy *u*. At this point, we introduce an additional stochastic element, assuming a certain distribution $f_U(u)$ of the actuator energy. Prior each attempt an actuator’s energy *u* is obtained by reverse sampling, according to: $u = F_{U}^{-1}(r)$, where *r* represents a uniformly distributed random number and $F_U$ the cumulative energy distribution.

**Fig. 10:**
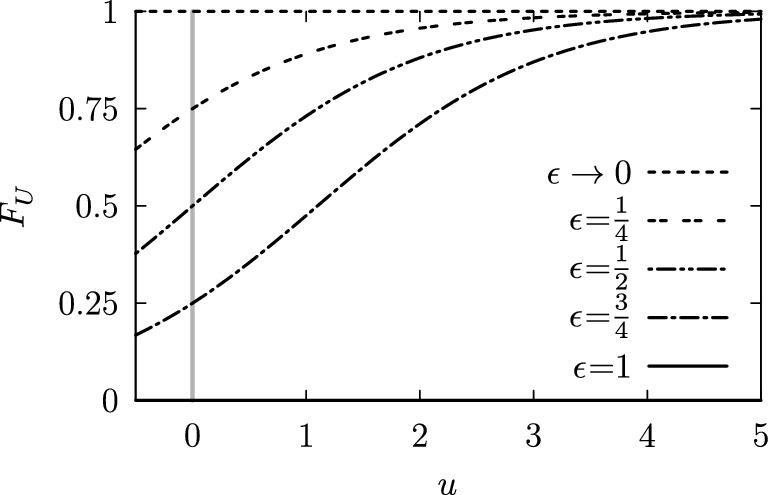
The curves show the actuators’ cumulative energy distribution $F_U(u)$ for different values of the energy parameter: $\epsilon \in \{\frac{1}{4}, \frac{1}{2}, \frac{3}{4}, 1 \}$ and for $\epsilon \rightarrow 0$

In the present simulations, we used the following expression:

2 $$\begin{aligned} F_U(u) = \left( \frac{\epsilon }{1-\epsilon }\exp (-u)+1\right) ^{-1}, \end{aligned}$$

where $u > 0$ and $\epsilon$ parameterizes the actuators’ energy supply. This function is illustrated in Fig. 10.

### Boolean network representation

In this study, the mucociliary dynamics on the epithelium are represented in terms of an adaptive Boolean network. Ciliated cells are imported in terms of Boolean actuators and represent the nodes of the network. The links between nodes represent mucociliary interactions, which we formulate in terms of Boolean functions.

Formally, a Boolean network consists of *N* elements $\{\psi _1, \psi _2, \ldots , \psi _N\}$, each of which is a binary variable $\psi _i \in \{0,1\}$ representing a node in the network. In general (Aldana et al. 2003), the value of a node $\psi _i$ at time $t+1$ is given as a function $f_i$ of its $K_i$ controlling elements at time *t*:

3 $$\begin{aligned} \psi _i^{t+1} = f_i(\psi _{{j_1}(i)}^t, \psi _{{j_2}(i)}^t, \ldots , \psi _{{K_i}(i)}^t)\,. \end{aligned}$$

The Boolean function $f_i$ as well as the number of controlling elements $K_i$ may be different for each node. The dependence on node *i* explicitly denotes that the set of controlling nodes with indices $\{j_1,j_2,\ldots ,K_i\}$ generally varies from one node to the other.

In our case, $\psi _{ij}$ at time $t+1$ is given as a function $f_{ij}$ of the local state configuration $\{\psi _{pq}^t\}_{pq \in n_{ij}^t}$ and the local distribution of mucus droplets $\{m_{pq}^t\}_{pq \in n_{ij}^t}$ at time *t*:

4 $$\begin{aligned} \psi _{ij}^{t+1} = f_{ij}\big ( \{\psi _{pq}^{t}\}_{pq \in n_{ij}^t}, \{ m_{pq}^t \}_{pq \in n_{ij}^t} \big ) \,. \end{aligned}$$

The term $n_{ij}^t$ denotes the set of index tuples, which identifies the set of all adjacent sites involved in the local interaction, and depends on the local state and mucus configuration at time *t*. The function $f_{ij}$ represents the local mucociliary interactions.

It is important to realize that the distribution of mucus droplets determines the topology of the network, as the update of mucus-free nodes is not affected by their local environment. As soon as an actuator gets occupied by a mucus droplet, it gets functionally connected to its neighboring nodes, as its subsequent state depends on the local state and mucus configuration. Consequently, in our network not only the network’s state is exposed to dynamics, but also the network’s topology, as the (re-)distribution of the droplets depends on the state transitions of the nodes and vice versa. Networks exhibiting such a feedback loop between the network’s state and its topology are called coevolutionary or adaptive networks (Gross and Blasius 2008).

### Concise formal model description

The state of the network is denoted by the two-dimensional Boolean state array $\varPsi ^{t} = \{\psi _{ij}^{t}\}$, where $i \in \{1, 2, \ldots , I\}$ denotes the row and $j \in \{1, 2, \ldots , J\}$ the column (Fig. 2). Therefore, $\varPsi ^{t}$ represents the collective state of *N* cells arranged along *I* rows and *J* columns at time *t*. Each cell $\psi _{ij}$ can be considered as a network node and either represents a ciliated cell, then $\psi _{ij}^t \in \{0,1\}$ (see Fig. 1), or an unciliated cell (two empty fields), then $\psi _{ij}^{t} =$ NaN (for $\forall t \ge 0$). The oscillating motion of cilia is imported by the permanent attempt of a ciliated cell to reverse its state. The state of the array of cells $\varPsi ^{t}$ is at any time associated with the distribution of mucus droplets, which is denoted by the array **M**$^t=\{m_{ijk}^t\}$, with $m_{ijk}^t \in {\mathbb {N}}_{0}$ representing the number of mucus droplets located on the field $k \in \{0,1\}$ of a site *ij*. After having initialized the Boolean state array $\varPsi ^0$ and its associated mucus distribution **M**$^0$, the temporal evolution of the set of *N* actuators, as well as of its associated mucus distribution, is achieved by sequentially updating the actuators as well as their mucus load. The state and mucus load of a Boolean actuator at time $t+1$ is given as a function of its own state and mucus load as well as of the state and mucus load of its adjacent actuators at time *t*. This function imports the coarse-grained local hydrodynamic coupling between ciliated cells. The update of the state of a cell *ij* can be formulated as:

5 $$\begin{aligned} \psi _{ij}^{t+1} = \left\{ \begin{array}{ll} \text {XOR}\{\psi _{ij}^t, c_{ij}^t\}, &{} {\text {if}}\; \psi _{ij}^t \in \{0,1\} \\ \text {NaN}, &{} \text {if}\,\, \psi _{ij}^t=\text {NaN}\,. \end{array}\right. \end{aligned}$$

The XOR function imports the actuators’ permanent attempt for state reversal, which represents the collectively synchronized oscillatory ciliary motion within a cell. The term $c_{ij}^t$ represents the local coupling among the nodes. If $c_{ij}^t$ gets true, the actuator’s state is reversed, i.e., $\psi _{ij}^{t+1} = \overline{\psi _{ij}^t}$, and in case $c_{ij}^t$ gets false, the actuator remains in its current state, i.e., $\psi _{ij}^{t+1}=\psi _{ij}^t$.

The local coupling term $c_{ij}^t$ is given as:

6 $$\begin{aligned} c_{ij}^t = \text {NOR}(l_0,l_1) \cdot \varTheta \left[r_{ij}^t-F_U\left( \Delta w_{ij}^t\right) \right]\,. \end{aligned}$$

$\varTheta (x)$ denotes the Heaviside step function, such that $\varTheta (0)=1$. The first term $\text {NOR}(l_0,l_1)$ in Eq. (6) imports a condition, which makes sure that an actuator can move in neither of the two possible “locked configurations” denoted by $l_0$ and $l_1$ (Fig. 8). For the choice of the square-lattice alignment $l_0$ and $l_1$ are formally given as:

7 $$\begin{aligned} \begin{aligned} l_0&= \text {AND}\left[ \varTheta \left( m_{ij}^t-1\right) , \overline{\psi _{ij}^t},\psi _{ij-1}^t,\overline{\psi _{i+1j}^t}, \psi _{ij+1}^t \right] \\ l_1&= \text {AND}\left[ \varTheta \left( m_{ij}^t-1\right) , \psi _{ij}^t,\overline{\psi _{ij-1}^t},\psi _{i-1j}^t, \overline{\psi _{ij+1}^t} \right] \end{aligned} \end{aligned}$$

If the considered actuator *ij* is not situated in a locked configuration (NOR$(l_0,l_1)=1$), it can invert its state, if its inversely sampled energy $u_{ij}^t=F_U^{-1}(r_{ij}^t)$, with $r_{ij}^t$ being a uniformly distributed random number $r_{ij}^t \in [0,1)$, is at least as large as $\Delta w_{ij}^t$, which denotes the amount of energy required to push the mucus load $m_{ij}^t$ away (see Fig. 7). Prior to each attempt an actuators’ energy $u_{ij}^t$ was obtained by reverse sampling according to the following cumulative energy distribution:

8 $$\begin{aligned} F_U(u) = \left\{ \begin{array}{ll} \left( \frac{\epsilon }{1-\epsilon } \exp (-u)+1 \right) ^{-1}, &{}{\text {for}} \;\forall u > 0\, \\ 0, &{}{\text {for}}\;u = 0, \end{array}\right. \end{aligned}$$

where the parameter $\epsilon \in [0,1]$ parameterizes the actuators’ energy. Therefore, the term $\varTheta \left[ r_{ij}^t-F_U(\Delta w_{ij}^t) \right]$ imports an “energetic condition” for an actuator not being situated in a locked configuration (NOR$(l_0,l_1)=1$) to move, which is subject to stochasticity. The term $1-F_U(\Delta w_{ij}^t)$ can be considered as the situation-specific moving probability.

Prior to each attempt of an actuator to move, we allow the mucus droplets to relax by invoking the maximum entropy principle. The mucus droplets are therefore most uniformly distributed over all locally involved adjacent fields maximizing the multiplicity, which is achieved by minimizing the denominator of the adopted multinomial coefficient:

9 $$\begin{aligned} W_n=\text {max}\left( M! \,/ \prod _{pq\in n_{ij}^t} m_{pq}^t! \right) , \end{aligned}$$

with $M = \sum _{pq\in n_{ij}^t} m_{pq}^t$ and the constraint: $|m_{pq}-m_{p^{\prime }q^{\prime }}|\le 1$, for $p\ne p^{\prime }\vee q\ne q^{\prime }$. The term $n_{ij}^t$ denotes the set of index tuples, which identifies the set of all adjacent sites, which are involved in the local interaction. The formal denotation of $n_{ij}^t$ is somewhat complicated and reads as follows (see also Fig. 9):

10 $$\begin{aligned} n_{ij}^t = \left\{ \begin{array}{ll} \{ (i+1,j) : \psi _{i+1,j}^t \in \{1,\text {NaN}\}, (i,j+1):\psi _{i,j+1}^t\in \{ 0, \text {NaN}\}, (i,j-1): \psi _{i,j-1}^t \in \{ 0, \text {NaN} \} \}, &{} {\text {if}}\;\psi _{ij}^t=0 \\ \{ (i,j+1) : \psi _{i,j+1}^t \in \{1,\text {NaN}\}, (i-1,j) : \psi _{i-1,j}^t \in \{0,\text {NaN}\}, (i,j-1) : \psi _{i,j-1}^t \in \{1,\text {NaN}\} \}, &{} {\text {if}}\;\psi _{ij}^t= 1 \\ \{ (i+1,j) : \psi _{i+1,j}^t \in \{1,\text {NaN}\}, (i,j+1), (i-1,j) : \psi _{i-1,j}^t \in \{0,\text {NaN}\}, (i,j-1) \}, &{} {\text {if}}\;\psi _{ij}^t= \text {NaN}\,. \end{array}\right. \end{aligned}$$

The amount of energy required for pushing the mucus load $m_{ij}^t$ away, which is denoted by $\Delta w_{ij}^t$, was determined according to:

11 $$\begin{aligned} \Delta w_{ij}^t = ln(W_{n} / W_{n-1})\,. \end{aligned}$$

$W_{n-1}$ denotes the multiplicity after the actuator *ij* would have cleared itself from the mucus load $m_{ij}^t$, which would therefore relax over $\# n_{ij}^t-1$ actuators. $W_{n-1}$ is consequently given as:

12 $$\begin{aligned} W_{n-1}=\text {max}\left( M! \,/ \prod _{pq\in n_{ij}^t} m_{pq}^{t+1}! \right) , \end{aligned}$$

where $m_{ij}^{t+1} = 0$ and $\psi _{ij}^t\in \{0,1\}$. Equation (12) implicitly specifies the update rule for the local mucus distribution, i.e., $m_{pq}^{t+1}$ with $pq \in n_{ij}^t$, which is subject to stochasticity and fully specified by Eq. (12), or Eq. (9) in case that $u_{ij}^t < \Delta w_{ij}^t$, with the constraints: $|m_{pq}^{t+1}-m_{p^{\prime }q^{\prime }}^{t+1}|\le 1$ (for $p\ne p^{\prime }\vee q\ne q^{\prime }$) and $M = \sum _{pq\in n_{ij}^t} m_{pq}^t = \sum _{pq\in n_{ij}^t} m_{pq}^{t+1}$ .

## Simulations and results

### Initial state

Prior to simulation, the state of the virtual epithelium has to be initialized. First, an array of *N* actuators with random states is generated, i.e., $\varPsi ^0$, where $\psi _{ij} \in \{0,1\}$ are uniformly distributed. Second, as the fraction *f* represents the proportion of unciliated cells, $f\cdot N$ randomly chosen sites are set to NaN. A certain amount of mucus droplets is then seeded on a randomly chosen site (uniformly distributed). For the sake of comparability between open and toric boundary conditions, the amount of mucus droplets was held constant during the simulation. Therefore, when applying open boundaries the mucus droplets leaving the modeling area were refed on a randomly chosen site. This way, we principally assumed the mucus excretion to be proportional to the mucus transport, which would of course need some kind of internal regulation mechanism in a real trachea.

### Parameter study

In order to assess under which circumstances our model self-organizes toward a self-cleaning virtual epithelium and how it reaches its properly functioning states dynamically, the influence of the model parameters, introduced in the previous chapter, on the network dynamics has been studied. The main parameter study encompasses the variation of the six model parameters in the following ranges. All cell alignments (USL, UHL, BHL, BHL + L), all update schemes (DAU, RAU1, RAU2, SRAU1, SRAU2) and all boundary conditions (OP, HC, VC, TO) have been used. The amount of mucus lumps has been varied in the range of 0.5%, 1%, 2%, 4%, ..., 256% of the total number of actuators in the grid (an amount of 4% corresponds to $0.04 \cdot N$ mucus droplets). The amount of unciliated cells has been set to 0%, 5%, 10%, 15%, 20%, 25%, and finally, the energy parameter has been set to 0, 0.25, 0.5, 0.75 and 1. Consequently, our main parameter study encompasses 24,000 simulation runs, which have been iterated for $10^5$ time steps using a grid size of $50 \times 50$ cells. These simulations can be seen as a starting point of further simulations we conducted. Each simulation run is characterized by its corresponding parameter setting. A specific parameter set is denoted as a combination of the form: (grid size, cell alignment, update scheme, boundary condition, mucus amount, energy parameter, amount of unciliated cells).

### Observables

**Fig. 11:**
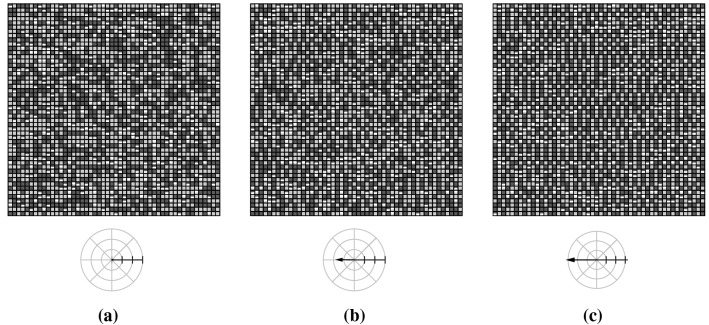
The panels show the network state of an exemplary simulation run at three different stages of the self-organization process. The crosshairs in the second row visualize the mucus transport velocity $\mathbf {v_g}(t^{\prime })$ for each stage (the radial tick interval corresponds to 0.2 (cells/it)). **a** Initial network state: randomly distributed states. **b** Network state after 100 iterations: due to the local interactions the emergence of order becomes visible. **c** After 1500 iterations: the self-organized cooperative behavior of actuators forms spatiotemporal patterns and exhibits efficient self-organized transport

To provide a qualitative impression of the self-organizing character of the network model and to illustrate the meaning of the chosen observables, we present first a typical simulation run. The parameters have been set to ($50 \times 50$, USL, DAU, TO, 16%, 0.5, 0%). Figure 11 shows the state of the network at three different stages of the self-organization process. Figure 11 represents a substitute for the temporal evolution of the network’s state, which is best visualized by the Online Resource 1. Actuators are colored in dark gray if $\psi _{ij}^t=1$ or in bright gray if $\psi _{ij}^t=0$. Mucus lumps are shown in white. Figure 11a shows the initial network state displaying the randomly generated initial configuration of states and mucus lumps. Figure 11b visualizes the state of the network after 100 iterations, representing an intermediate stage of the self-organization process, as the emergence of global order becomes clearly visible. Finally, Fig. 11c shows the network state after 1500 iterations. At this stage, the fascinating self-organization process has almost completed, and the actuators finally behave strongly coordinated at a global scale.

By examining the successive network states in movie_S1 (Online Resource 1), one can observe that the particles get initially moved around disorderly. Quickly, the actuators start to cooperate by adjusting their oscillations to the oscillations of the surrounding actuators until the particles get efficiently transported into a well-defined direction—what we call self-organized transport. Consequently, in our models the self-organization process is actually twofold. As in accordance with the emergence of spatiotemporal patterns, the virtual epithelium exhibits self-organized transport. This coevolution of the network state and its associated mucus transport has been quantified in terms of several observables, which are introduced in the following.

#### Mucus transport velocity

For each actuator at the position *ij* at time *t*, we assign a local mucus velocity in terms of the local displacement of the center of mass (CM), which we denote as $\mathbf {v}_{ij}(t)$ and has been calculated (if $m_{ij}^t > 0$) according to:

13 $$\begin{aligned} \mathbf {v}_{ij}(t) \doteq \frac{\sum _{pq \in n_{ij}^t} (\mathbf {r}_{ij} - \mathbf {r}_{pq}) (m_{pq}^{t+1} - m_{pq}^t)}{\sum _{pq \in n_{ij}^t} m_{pq}^t} \end{aligned}$$

$\mathbf {v}_{ij}(t)$ measures the redistribution of mucus droplets in the neighborhood $n_{ij}^t$ of the activated actuator at *ij* in terms of the local displacement of the CM. $\mathbf {r}_{ij} - \mathbf {r}_{pq}$ denotes the distance vector pointing from the activated actuator at *ij* to the neighboring actuators at *pq*. This relative distance vector gets weighted by the redistributed mucus droplets $(m_{pq}^{t+1}-m_{pq}^{t})$.

In order to quantify the global transport velocity $\mathbf {v}_g(t^{\prime })$ (remind that $t^{\prime } \doteq t/N$), we calculate the mucus-weighted average over the whole array of actuators of the formerly defined local velocity of the CM in Eq.(13) according to:

14 $$\begin{aligned} \mathbf {v}_g(t^{\prime }) \doteq \frac{\sum _{ij} \mathbf {v}_{ij}(t^{\prime }) \sum _{pq \in n_{ij}^t} m_{pq}^{t^{\prime }}}{\sum _{ij}\sum _{pq \in n_{ij}^t} m_{pq}^{t^{\prime }}}\,. \end{aligned}$$

$\mathbf {v}_g(t^{\prime })$ represents an approximation to the actual average mucus transport velocity, as droplets moving more than one field in one single time step are neglected. As these movements only happen due to fluctuations originating from mucus relaxation, $\mathbf {v}_g(t^{\prime })$ represents a good measure for the area-averaged mucus transport velocity.

**Fig. 12:**
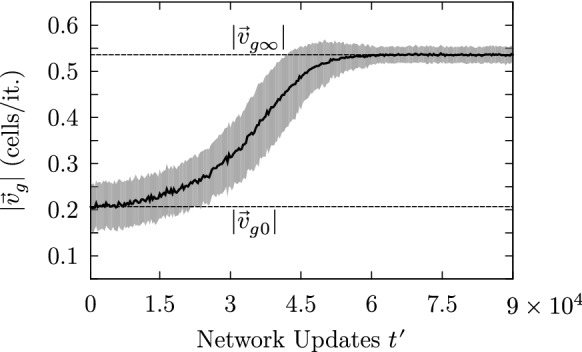
Temporal evolution of the average mucus transport speed of an ensemble consisting of 100 simulation runs. The typical limited growth behavior was omnipresent in all of our simulation runs displaying a self-organizing behavior

Figure 12 shows the temporal evolution of the global mucus transport speed $|\mathbf {v}_g(t^{\prime })|$ for an ensemble consisting of 100 simulation runs differing only by their initial state. Each ensemble member corresponds to a simulation run for which the parameter setting ($50 \times 50$, BHL + L, RAU1, OP, 10%, 1, 0%) has been used. The curve shows the temporal evolution of the ensemble mean (solid line) and its standard deviation (shaded area) and displays the typical saturation-like behavior of the average mucus transport speed, which has been observed for each parameter setting showing a self-organizing behavior. Accordingly, we can define the initial mucus transport velocity $\mathbf {v}_{g0} \doteq \lim \limits _{t^{\prime }\rightarrow 0}\mathbf {v}_g(t^{\prime })$ and the final mucus transport velocity $\mathbf {v}_{g\infty } \doteq \lim \limits _{t^{\prime }\rightarrow \infty }\mathbf {v}_g(t^{\prime })$. Consequently, the global average transport velocity can be expressed as

15 $$\begin{aligned} \mathbf {v}_g(t^{\prime }) = \mathbf {v}_{g0} + (\mathbf {v}_g(t^{\prime }) - \mathbf {v}_{g0}) \doteq \mathbf {v}_{g0} + \Delta \mathbf {v}_g(t^{\prime }), \end{aligned}$$

where $\Delta \mathbf {v}_g(t^{\prime })$ can be seen as the effectively self-organized mucus transport velocity.

#### Mucus transport direction

We observed that some parameter sets drive the model toward well-organized network states exhibiting non-negligible area-averaged transport speeds. However, some of these well-organized states exhibit unrealistic velocity fields.

**Fig. 13:**
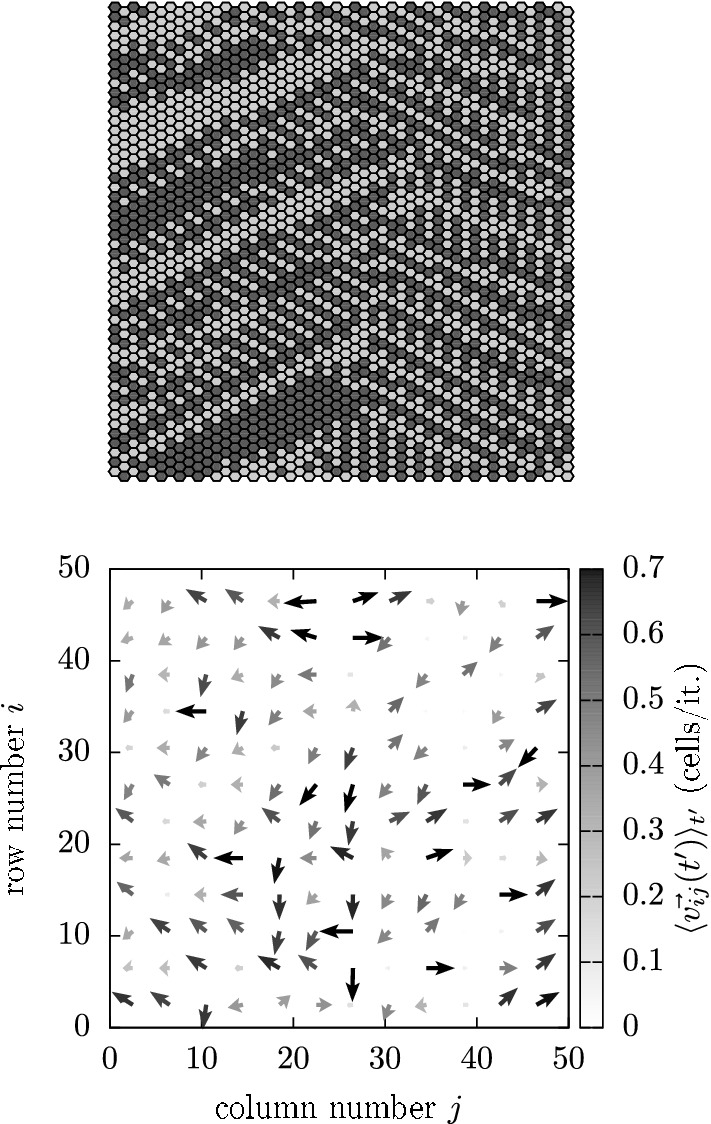
Example of a highly ordered network state (top) displaying a disordered (temporally averaged) velocity field (bottom). The gray level indicates the droplets’ speed (cell/it.)

The network state shown in Fig. 13

(upper panel), and its corresponding (temporally averaged) velocity field (lower panel) has been generated by applying the parameter set ($50 \times 50$, UHL, DAU, OP, 64%, 1, 0%). It can be seen that even if the network state is well organized the corresponding velocity field appears to be rather disordered. A closer look reveals that the network state as well as its corresponding velocity field is divided into two parts. The right half transports the mucus with a tendency to the right, while the transport on the left half tends to the left. The global average velocity amounts to $\mathbf {v}_{g\infty } = (-\,0.14, -\,0.03)$ cells/it.

In order to classify parameter sets generating such odd velocity fields as malfunctioning, we measured the spread of the (temporally averaged) local velocity fields in terms of $\langle \cos \theta \rangle \doteq \langle \cos \theta _{ij}\rangle _{ij}$, where $\langle \cdots \rangle _{ij}$ indicates spatial averaging. We defined $\cos \theta _{ij}$ as:

16 $$\begin{aligned} \cos \theta _{ij} \doteq \lim \limits _{t^{\prime } \rightarrow \infty }\frac{ \langle \mathbf {v}_{ij}(t^{\prime }) \rangle _{t^{\prime }}\cdot \langle \mathbf {v}_g(t^{\prime })\rangle _{t^{\prime }} }{|\langle \mathbf {v}_{ij}(t^{\prime })\rangle _{t^{\prime }}| \cdot |\langle \mathbf {v}_{g}(t^{\prime })\rangle _{t^{\prime }} |}, \end{aligned}$$

where $\langle \cdots \rangle _{t^{\prime }}$ indicates temporal averaging (over the last $10^3$ iterations of each simulation). For the velocity field shown in Fig. 13$\langle \cos \theta \rangle$ amounts to 0.18 indicating not properly directed transport.

#### Transient time

**Fig. 14:**
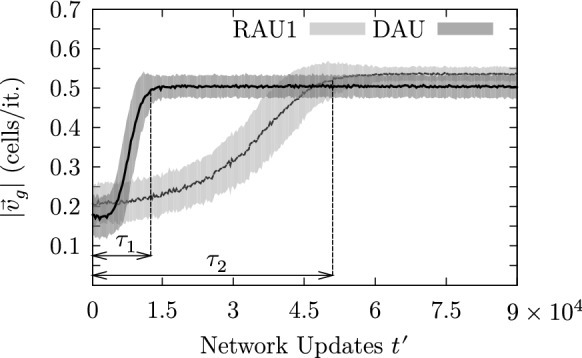
The curves show the temporal evolution of the average mucus speed for two ensemble simulations differing only by their update scheme. The darker and brighter curves correspond to simulations for which DAU and RAU1 have been applied, respectively. Simulations initialized with the update scheme DAU exhibit an accelerated self-organization reaching saturation roughly four times faster than those simulations initialized with RAU1, which is indicated by the corresponding transient times $\tau _1$ and $\tau _2$

Figure 14 depicts the temporal evolution of two ensembles consisting of 100 ensemble members differing only by their initial condition. The brighter band corresponds to the curve presented in Fig. 12 showing the evolution of the average mucus transport speed being generated with the parameter setting ($50 \times 50$, BHL + L, RAU1, OP, 10%, 1, 0%). The darker curve has been generated with exactly the same parameter settings apart from the update scheme. Instead of RAU1, the update DAU was used. Both ensembles show the typical saturation-like temporal evolution of the average transport speeds, which reflects the capturing of the system dynamics. We calculated the transient time based on the transient behavior of the average transport speed, which roughly follows an exponential behavior: $|\mathbf {v}_g(t^{\prime })| = |\mathbf {v}_{g0}| + \exp (-t^{\prime }/\tau ') \cdot (|\mathbf {v_{g\infty }}| - |\mathbf {v_{g0}}|)$. The transient time $\tau$ has been defined as $\tau \doteq 3\cdot \tau '$. As one can notice in Fig. 14, the transient time for the ensemble simulation using the deterministic update scheme DAU is roughly four times shorter than the one for which the update scheme RAU1 was used. This behavior may be more fundamental and shall be discussed later.

#### Autocorrelation

**Fig. 15:**
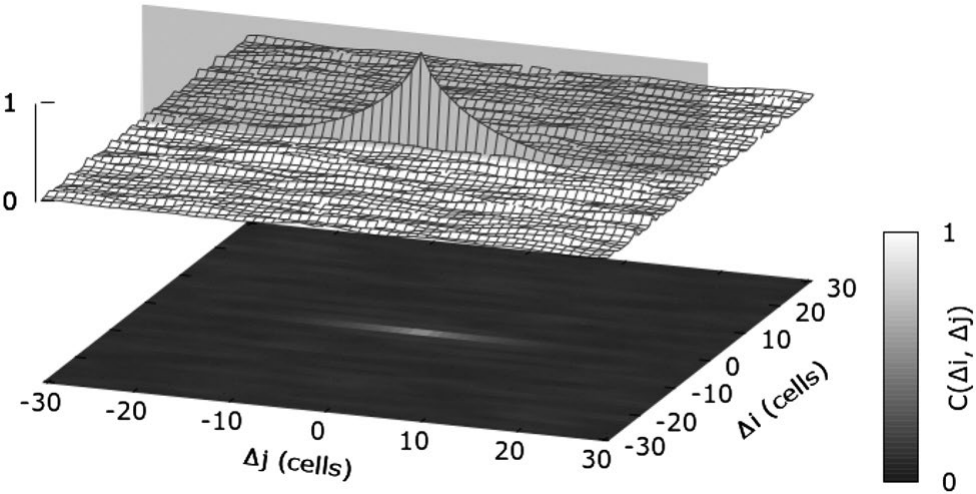
The autocorrelograms of self-organized network states typically display an elongated shape along a certain direction (indicated by the bright gray vertical plane), along which we determined the autocorrelation length $\rho _c$

In order to characterize the coordination among the actuators in a particular network state, we use the spatial autocorrelation function C$(\Delta i, \Delta j, t^{\prime })=\sum \limits _{i,j} \varPsi (i,j,t^{\prime }) \varPsi (i+\Delta i,j+\Delta j,t^{\prime })$, where $\Delta i$ and $\Delta j$ denote the shifts into the *i*- and *j*-direction, respectively. As we would like to compare different simulation runs with respect to the emergent spatial order, we used the autocorrelogram to determine the spatial autocorrelation length $\rho _c$, representing a measure for the degree of order in a network state. As most correlograms appear to be strongly elongated as illustrated in Fig. 15, we determined the autocorrelation length along the direction of maximum correlation, which is indicated by the gray plane in Fig. 15. Note that in roughly 95% of all self-organized properly self-cleaning states, the direction of maximum correlation coincides with the direction of mucus transport. As most correlograms show an exponential-like decrease along the direction of maximum correlation, the autocorrelation length $\rho _c$ has been determined according to:

17 $$\begin{aligned} \rho _c(t^{\prime }) = \text {max}\left\{ \frac{1}{2} \sum \limits _{v=v_1\ldots v_2} \text {C}(v, \gamma (v), t^{\prime }) \right\} \end{aligned}$$

Equation (17) delivers the autocorrelation length $\rho _c$ along the line of maximum correlation, which is represented by the parameterization $\gamma (v)$.

### Coevolution of spatiotemporal patterns and transport

**Fig. 16:**
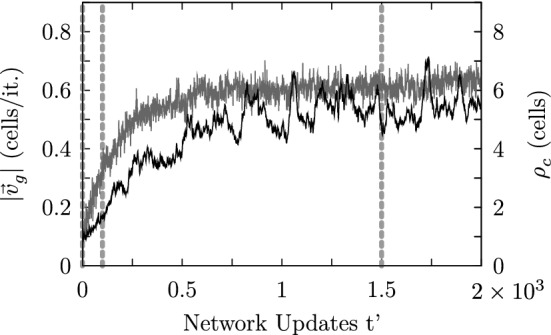
The graph illustrates the coevolution of the autocorrelation length of the network state along the direction of maximum correlation $\rho _c(t^{\prime })$ (dark curve) and the area-averaged mucus transport speed $|\mathbf {v_g}(t^{\prime })|$ (bright curve). The vertical dashed lines indicate the initial ($t^{\prime }=0$), an intermediate ($t^{\prime }=100$) and a final stage ($t^{\prime }=1500$) of the self-organization process

The coevolution of self-organized transport and spatiotemporal patterns is illustrated in Fig. 16. The brighter curve shows the area-averaged transport speed $|\mathbf {v_g}(t^{\prime })|$ (according to Eq. (14)) illustrating the build up of self-organized transport. The darker curve represents the autocorrelation length of the network’s state at time $t^{\prime }$. As at the beginning of the simulation the mucus droplets get displaced almost erratically, the corresponding area-averaged transport speed almost vanishes. As time passes, the average particle speed grows until the coordination of the actuators reaches a maximum. The three vertical dashed lines indicate the moments at which the three network states shown in Fig. 11 have been recorded. Interestingly, all model settings leading to self-organized transport have shown a similar growth behavior with respect to the global average transport speed as the one depicted in Fig. 16. This typical transient dynamical behavior reflects the restricted growth of the cooperation among the actuators. This sigmoid logistic-like dynamical behavior can be observed in other studies investigating self-organizing processes as well and appears to be omnipresent for self-organizing processes. An example is provided by Fig. 2 in Niedermayer et al. (2008), in which the self-organized synchronization and wave formation in one-dimensional cilia arrays has been studied.

Our model typically exhibits a coevolution of the network’s state and its associated mucus transport. As indicated earlier, we suggest to see this twofold self-organization in the context of adaptive Boolean networks. Since mucus droplets functionally connect an actuator to its surrounding and disconnect an actuator when squeezed away, the network topology is exposed to dynamics and interrelated to the dynamics of the network’s state. Consequently, we observe not only dynamics *on the network*, represented by state transitions, but also *dynamics of the network*, caused by the transportation of the mucus droplets. This means that changes in the network’s state are affected by the network’s topology and vice versa, forming a feedback loop between the topology and the state of the network. The resulting coevolution of the network state and the network topology represents the characteristic property of coevolutionary or adaptive (Boolean) networks (Gross and Blasius 2008; Rohlf and Bornholdt 2009).

### Characteristics of attractor states

#### Attractor states

Since $\varPsi ^{t^{\prime }}$ represents the state of the Boolean network at time $t^{\prime }$ containing *N* Boolean variables, the set of all 2$^N$ possible network states forms the state space of the Boolean network. The successive network states $\varPsi ^0,\varPsi ^1, \varPsi ^2 \ldots$ form a trajectory in the state space. In the case of deterministic Boolean networks, a network sooner or later reaches a state, which has been reached before (due to the finite state space) and consequently enters a cycle consisting of a subset of states of the state space, which is called an attractor. A more general definition for dynamical systems says that an attractor is a set of states to which the system evolves after a long enough time (Greil 2009). The transient time $\tau$ is the number of states a network undergoes (starting from an initial state), before it reaches an attractor (Wuensche 1998; Gershenson 2004a; Greil 2012).

We used the term “attractor” according to the following definition: as soon as the successive network states $\varPsi ^{t^{\prime }}$ have started to revisit a small subset of states of the state space (and therefore, $t^{\prime }\ge \tau$), we say that the network has reached an attractor. In this definition of the term “attractor,” we therefore only consider the state of the network nodes $\varPsi$, i.e., the collective state of the actuators, while the distribution of the mucus droplets *M* was not taken into account. Due to the coevolutionary character of the network, the (re-)distribution of the mucus droplets reaches, however, another kind of “attractor.” The exclusion of the (re-)distribution of mucus droplets allowed to detect deterministic attractors, i.e., a deterministic succession of states $\varPsi ^{t^{\prime }}$ (with $t^{\prime }\ge \tau$, where $\tau$ denotes the transient time) the network cyclically undergoes, while the trajectories of the (re-)distribution of the mucus droplets continue to be subject to local stochasticity in these (state) attractors.

Within the meaning of the state space concept, the curves in Fig. 16 can be seen as the network’s transient behavior. The saturation-like behavior reflects the capture of the network dynamics by an attractor. Accordingly, the strongly ordered network state shown in Fig. 11 represents an attracting state.

#### Transport of attractor states

**Fig. 17:**
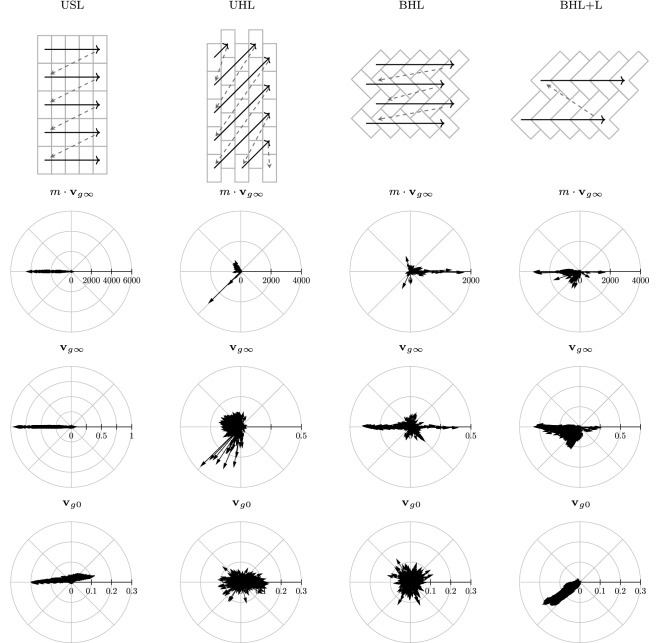
The rows present the distribution of the 24,000 mucus transport vectors $m\cdot \mathbf {v}_{g\infty }$ (#m$\cdot$cells/it.), average terminal velocities $\mathbf {v}_{g \infty }$ (cells/it.) and the initial velocities $\mathbf {v}_{g0}$ (cells/it.) for each alignment (column-wise)

Figure 17 presents an overview of the terminal mucus transport ($m\cdot \mathbf {v}_{g\infty }$), the terminal mucus transport velocity ($\mathbf {v}_{g\infty }$) and the initial mucus transport velocity ($\mathbf {v}_{g0}$) for each cell alignment (column-wise). The first row indicates to which cell alignment each column corresponds to and illustrates the sequences after which the actuators have been activated when the deterministic update scheme DAU has been applied. Apparently, the globally averaged terminal transport velocities have a clear preference considering their direction for each cell alignment. Generally, the preferred transport direction seems to be oppositely-oriented to the direction of the deterministic update signal. However, for a small set of the settings using BHL or BHL + L the transport shares the direction of the update signal.

#### Classification attempt

**Fig. 18:**
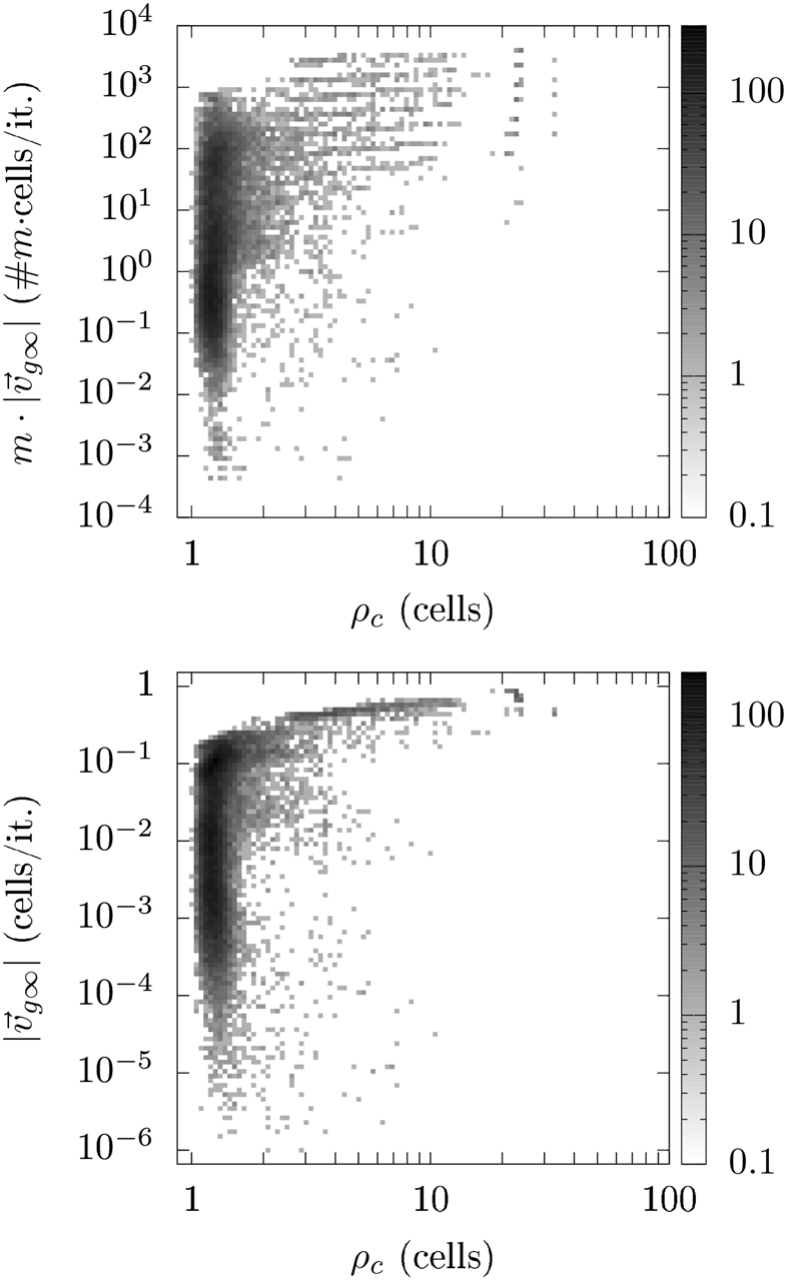
Distribution of the frequency densities of attracting states considering their transport rate (top) and transport speed (bottom) with respect to their corresponding correlation length

Figure 18 illustrates how the transport capability (upper colormap) and the transport speed (lower colormap) of attractor states are related to the correlation length. The gray scale indicates the frequency density.

Considering the attractors’ function and structure a rough classification into the following four classes (C1–C4) seems appropriate: C1: nontransporting disorganized states, C2: transporting disorganized states, C3: nontransporting structured states and C4: transporting structured states. We measured the transport capability in terms of the transport speed $|\mathbf {v_{g\infty }}|$ and the degree of the organization of the expression patterns in terms of $\rho _c$. States/settings exhibiting a higher $|\mathbf {v}_{g\infty }|$ than 0.1 (cells/it.) are considered as transporting, while states/settings exhibting a $|\mathbf {v}_{g\infty }|$ of less than 0.01 (cells/it.) have been considered as nontransporting. States satisfying $\rho _c > 2$ (cells) have been classified as organized and states with a $\rho _c$ of less than 1.5 (cells) as disorganized ($\rho _c$ of randomly generated initial states were shorter than 1.6 cells).

Here, we just note that attractor states of class 4 still contain many states exhibiting odd velocity fields, like bisected or disordered ones, which still reach considerable terminal transport speeds. Consequently, only a subset of class 4 is considered as “properly self-cleaning” attractor states, whose velocity fields shall fulfill further conditions specified in the next section.

#### Effectively self-organized properly self-cleaning attractors

In order to find the parameter values allowing the network to self-organize toward properly self-cleaning attractor states, we finally classified the settings as “functioning” or ”malfunctioning.” A certain parameter set is classified as functioning, if the following four conditions are simultaneously fulfilled after an integration time of $t^{\prime }=10^5.$ (1) The globally averaged transport speed $|\mathbf {v}_{g\infty }|$ is faster than 0.1 cells/it., which would roughly correspond to 20 μm/s in the real system (assuming a ciliary beat frequency of 10 Hz and the diameter of a ciliated cell being 10 μm). (2) We require the velocity field being sufficiently directed by: $\langle \cos \theta \rangle > 0.65.$ (3) The velocity field must be self-organized and not simply being imposed by the choice of a parameter set leading to an initial tendency of the transport direction. Consequently, by requiring $|\Delta \mathbf {v}| \doteq |\mathbf {v_{g\infty }}-\mathbf {v_{g0}}| > 0.1,$ we demand that the velocity field changes (at least its direction) in the course of a simulation. (4) The autocorrelation length $\rho _c$ has to be longer than two cells. The number of iterations may be seen as a fifth condition considering the efficiency of the self-organization process. As thus, the transient time $\tau$ has to be shorter than $10^5$ iterations. The classification of parameter sets as functioning and malfunctioning was applied to the parameter study encompassing 24,000 parameter sets. 564 out of 24,000 settings ($\widehat{=} 2.4\%$) have been classified as functioning. Table 1 shows how these functioning settings spread across the different cell alignments and update schemes (values are listed in (%)). Table 1 summarizes the following observations. The cell alignment USL can cope with the largest set of parameter settings and makes up $84.2\%$ of all functioning settings. $98.2\%$ of all functioning settings are made up by settings using either the cell alignment USL or BHL + L. The deterministic update scheme DAU can cope with each cell alignment and $89.6\%$ of all functioning settings were generated by this update scheme. None of the settings using the completely random update RAU2 evolves the network to a self-organized self-cleaning state. The cell alignment BHL + L (lowest row in Table 1) can cope with all update schemes except the fully random update scheme RAU2. Apparently, a certain degree of local determinism is advantageous for an efficient self-organization toward properly transporting attractor states, since all functioning parameter settings either involve DAU or BHL + L.

**Table 1:** The table shows how the functioning states spread across the different topologies and update schemes

	DAU	RAU1	RAU2	SRAU1	SRAU2
USL	84.2	0.0	0.0	0.0	0.0
UHL	1.2	0.0	0.0	0.0	0.0
BHL	0.5	0.0	0.0	0.0	0.0
BHL + L	3.7	1.6	0.0	0.9	7.8

The values represent the corresponding proportions of all functioning states in (%)

#### Mean wave propagation direction in stereotypical functioning states

The set of functioning parameter values drives the model toward families, or stereotypes, of attractor states comprising very similar attractor states. In order to determine the mean direction of wave propagation in these stereotypical attractors, we employed the concepts outlined by Ryser et al. (2007) and examined sequences of sequential attracting states of one specific representative parameter set for each stereotype. Here, we would like to report the summarizing comparison between the direction of the mean wave propagation and the direction of transport, what can be found in Table 2. Note that mean *k*-vectors visualized by double arrows indicate that the mean direction of wave propagation could not be determined unambiguously, which comes from the Boolean nature of the model (undersampling).

**Table 2:**
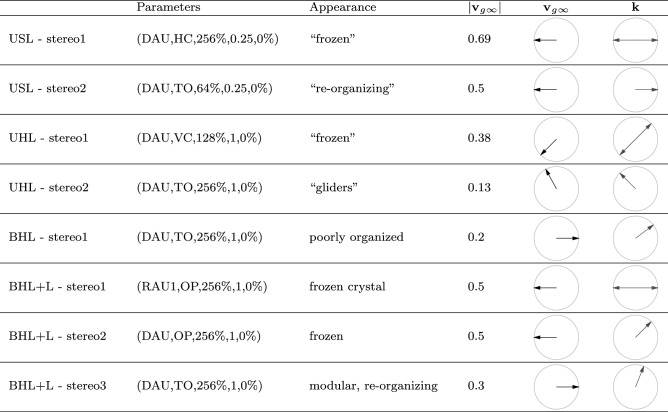
The table reports the parameter sets of stereotypical representatives, the typical appearance of attracting states, the transport speed and particularly, the direction of transport as well as of the mean wave propagation is visualized

### Dynamical characteristics

#### Imposed asymmetry as a starting assistance for self-organized self-clearing

**Fig. 19:**
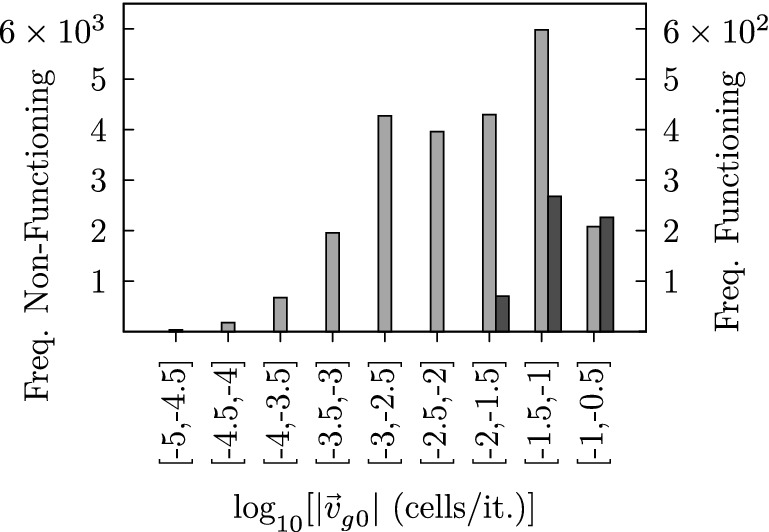
The histograms depict the frequency distributions of functioning (dark gray bars) and malfunctioning (bright gray bars) parameter sets considering their respective values of $|\mathbf {v}_{g0}|$

In this section, we discuss the potential role of imposed asymmetries as a control parameter. As shown in Table 1 functioning settings either include DAU or BHL + L, or both—all other settings do not result in self-organized self-clearing states. Consequently, these settings may reveal a common characteristic which ultimately leads to self-organized self-cleaning. We realized that properly functioning parameter sets consistently impose an initial tendency of the transport direction. The initial area-averaged transport speed $|\mathbf {v}_{g0}|$ can be used in order to quantify the anisotropy of the transport at time $t^{\prime }=0$. In order to estimate the expected initial transport speed at $t^{\prime }=0$ of a certain parameter setting, we used a grid consisting of $1500 \times 1500$ actuators, which have been updated for a single time step ($\varPsi ^0 \rightarrow \varPsi ^1$). This has been done for an ensemble of 100 simulation runs. The ensemble mean provides an estimate for the imposed initial transport speed $|\mathbf {v}_{g0}|$. Figure 19 illustrates the frequency distribution of functioning (dark gray bars) as well as malfunctioning (bright gray bars) parameter sets considering their corresponding $|\mathbf {v}_{g0}|$-classes (10 logarithmically spaced $|\mathbf {v}_{g0}|$-classes between $10^{-5}$ and 1). Note that the scale of the two ordinates differs by one order of magnitude.

It can be particularly seen that the relative frequency of functioning parameter sets increases with increasing $|\mathbf {v}_{g0}|$-values. Moreover, all functioning parameter sets correspond to $|\mathbf {v}_{g0}|$-values being faster than 0.01 cells/it.

**Fig. 20:**
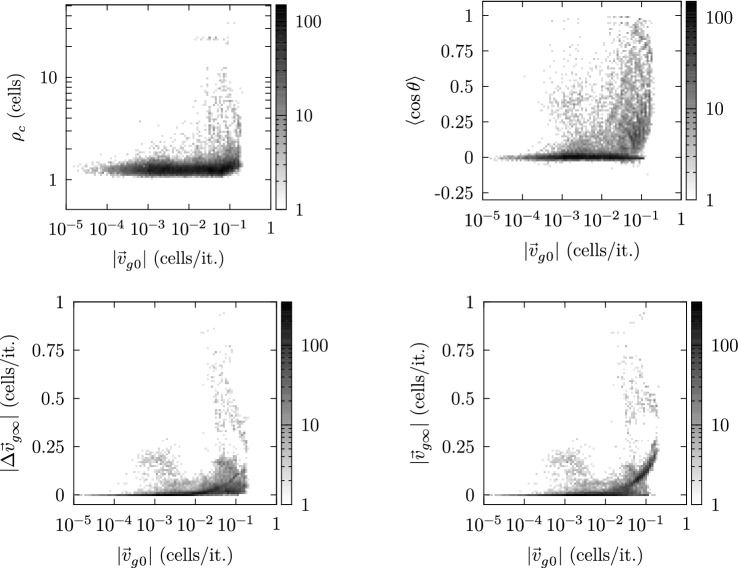
From top left to bottom right: frequency densities (grayscales) illustrating the tendency toward higher values for $\rho _c$, $\langle \cos \theta \rangle$, $|\Delta \mathbf {v}_{g\infty }|$ and $|\mathbf {v}_{g\infty }|$ for settings imposing larger values of $|\mathbf {v}_{g0}|$

Correspondingly, the measures $|\Delta \mathbf {v}|$, $\rho _c$, $\langle \cos \theta \rangle$ and $|\mathbf {v_{\infty }}|$ classifying the parameter sets tend to increase with increasing values of $|\mathbf {v}_{g0}|$, which is illustrated in Fig. 20. Consequently, Figs. 19 and 20 indicate that $|\mathbf {v}_{g0}|$ may trigger the model toward self-organized self-clearing.

**Fig. 21:**
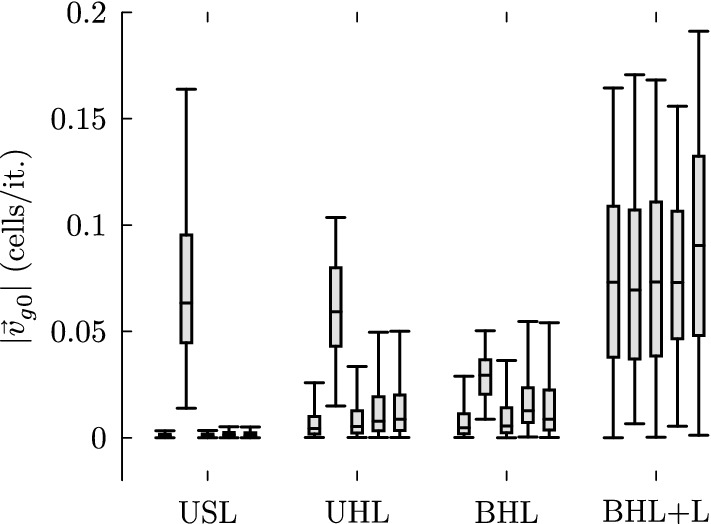
Box plot diagrams to visualize the effect of the choice of the cell alignment and the update scheme on the initial transport speed. The box plots of each cell alignment correspond to the respective update scheme, according to the order: RAU1, DAU, RAU2, SRAU1, SRAU2

The box plots shown in Fig. 21 (whiskers are set in order to indicate the range covered by 95% of all values) display how the $|\mathbf {v}_{g0}|$-values distribute over the different update schemes and cell alignments. One can see that the highest values for $|\mathbf {v}_{g0}|$ are reached by settings using DAU or BHL + L. This clearly suggests that update schemes with local determinism impose an asymmetry among the local interactions, what triggers efficient self-organization toward self-clearing states.

#### Effect of the update scheme on the network dynamics

**Fig. 22:**
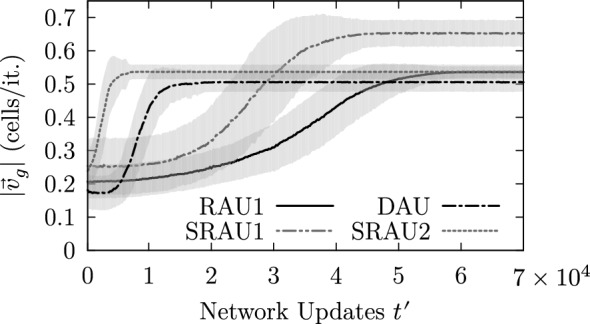
The curves depict the temporal evolution of the average transport speed for four ensembles corresponding to different update schemes for which the cell alignment BHL + L was chosen

**Fig. 23:**
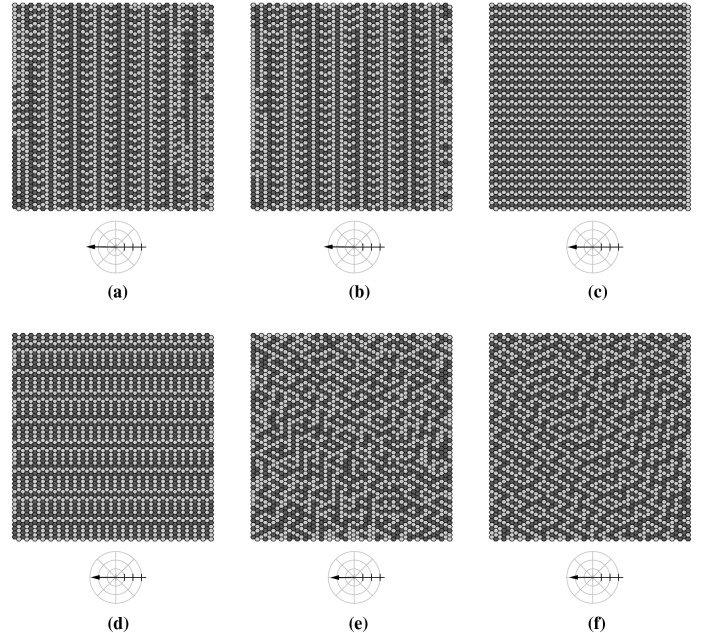
Examples of attracting states reached by applying four different update schemes to the BHL + L alignment. From top left to bottom right: attracting state reached by **a** SRAU1, **b** SRAU1, **c** RAU1, **d** SRAU2, **e** DAU, **f** DAU. For RAU1 and SRAU2 100 different initial states reached the same attracting network state shown in (**c**, **d**), respectively, whereas applying SRAU1 and DAU leads to a diversity of attracting network structures, which in contrast to the perfect regular structures generated by RAU1 and SRAU2, show “defects.” The crosshairs visualize the mucus transport direction and its magnitude of the corresponding attractors (the radial tick interval is set to 0.2 [cells/it.]), i.e., $\mathbf {v_{g,\infty }}$

**Fig. 24:**
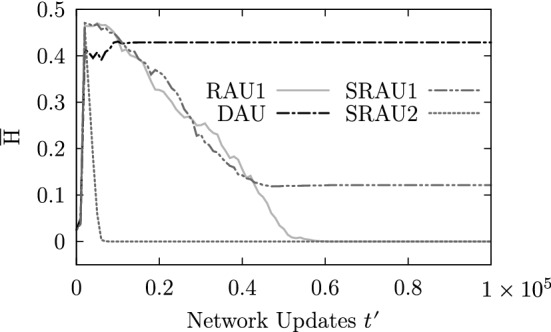
The curves show the average Hamming distance between the network state of a reference run and four simulation runs for which the initial state has been perturbed with respect to the reference run (1–4% of the nodes have been inverted)

As settings using the locally prescribed four-phase sequence (BHL + L) can cope with each update scheme (except with the complete random scheme RAU2), we compared the dynamic behavior produced by the different update schemes by simulating an ensemble of 100 simulation runs using the parameter settings: ($$50 \times 50$$,BHL + L, $$*$$, OP, 10%, 1, 0%), where $$*\in$$$$\{$$DAU, RAU1, SRAU1, SRAU2$$\}$$. Figure 22 shows the transient behavior for the average mucus transport speed of the four ensembles, which correspond to the different update schemes. Each setting produces a saturation-like behavior reflecting the capturing of the dynamics by attractors. As one can see, settings using SRAU2 and DAU reach their attractors considerably faster than settings using SRAU1 and RAU1. In order to characterize the state space corresponding to each setting, we counted the number of attractors and the attractor periods. It turned out that the four ensembles specified above, all reach cyclic deterministic attractors with a period of four, which result from the prescribed cyclic four-phase sequence. Furthermore, SRAU2 and RAU1 drive each of the 100 different initial conditions toward the same attracting state, which is shown in Fig. 23. DAU and SRAU1 produce more realistic dynamics, as the 100 simulation runs were driven toward 90 and 95 different attracting states, respectively. For DAU and SRAU1, two exemplary attracting states are depicted in Fig. 23. Even if SRAU1 drives different initial conditions toward different attracting states, the attracting states strongly resemble each other, as indicated in Fig. 23. DAU produces more different emerging structures. In order to verify this observation, we ran 5 simulation runs for each update scheme, which differed by their initial state. But this time we prescribed how much their initial state shall differ against a reference run. Namely, we switched the state for 1%, 2%, 3% and 4% of all actuators, respectively, and compared the networks’ state at each time step to the reference run in terms of the normalized Hamming distance^1^ (e.g., Greil 2009) see Fig. 24). The average Hamming distance corresponding to SRAU2 and RAU1 decreases to zero, which means that all states reach the same attractor. On the other hand, SRAU1 and DAU produce different attractors, but the attractors produced by DAU show an almost maximum Hamming distance. This behavior reflects the ability of the system to find new attractors in case of a perturbation and to conform to changes, which is an important characteristic for living systems. Consequently, if an attractor which has been reached by applying DAU gets perturbed, the system conforms to changes and runs into a new attractor.

#### Open boundaries: marginal nodes guiding the structure emergence

**Fig. 25:**
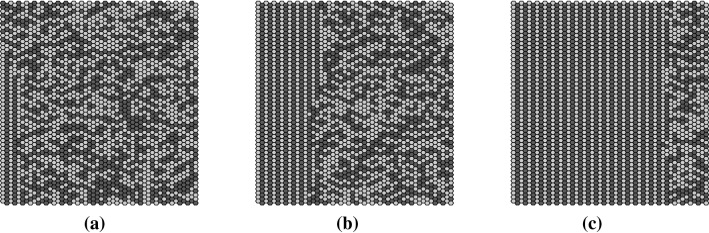
Three snapshots of the network’s state at three different stages of the self-organization process (time increases from **a**–**c**). The network states were generated by applying RAU1 to the BHL + L alignment with open boundary conditions. The structure emergence sets in at the left boundary and spreads to the right over the whole network

First of all we consider a completely dense carpet of ciliated cells represented by a parquet of actuators. In this “densely ciliated carpet case,” we observe that settings with open boundaries yield a structure emergence always starting at the same open boundary, from which it spreads over the whole network. This behavior is illustrated in Fig. 25, where three snapshots of the network state at three different stages of the self-organizing process are shown. The graphs have been generated by applying open boundaries, RAU1 and BHL + L. Further, we observed that settings leading to the “crystallization-process,” shown in Fig. 25, only show this self-organizing behavior as long as the boundary, from which the structure spreads, is open. This means that similar network dynamics have been observed for open and horizontal cylindrical boundaries. On the other hand, if we chose toric or vertical cylindrical boundaries, for which the “structure-triggering” border is glued to the opposing border, the model displays a completely different dynamical behavior with much lower self-organized transport speeds and much less well-ordered network states. The only exception is given by the settings using the square-lattice alignment, which shows self-organized directed transport under each boundary condition.

Online Resource 2, 3 and 4 illustrate the structure emergence from an open boundary. They have been generated by applying ($50 \times 50$, USL, DAU, OP, 10%, 0, 0%), ($50 \times 50$, BHL + L, DAU, OP, 15%, 1, 0%) and ($50 \times 50$, BHL + L, RAU1, OP, 15%, 1, 0%), respectively.

#### Leaders and followers

Since we observed for the “densely ciliated carpet case” when using open boundaries that the emergence of highly ordered structures always sets in from the same boundary, the actuators at the boundary seem to play an important role for the self-organizing process, and consequently, it appears that there exists a kind of hierarchy among the actuators.

**Fig. 26:**
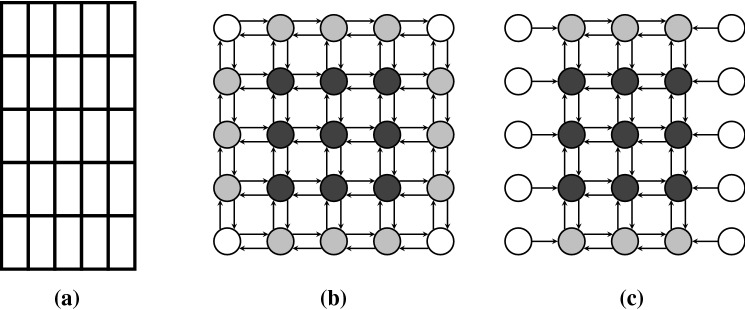
An array of $5 \times 5$ actuators aligned in the square lattice is shown in (**a**). Actuators represent the nodes of the network and local interactions the links. **b** Shows the network topology considering the exchange of mucus particles among nodes. **c** Illustrates the network topology considering the state updates. **b**, **c** Correspond to open boundary conditions. In (**c**), it can be seen that open boundaries introduce “leading nodes” at the margin

This hierarchy among the nodes is caused by the underlying network topology and is primarily characterized by the distribution of in- and out-degrees. In Fig. 26, we illustrate the underlying network topology for an array consisting of $5\times 5$ cells arranged in a square lattice assuming open boundaries. The middle panel in Fig. 26 illustrates the network topology considering the possible pathways of the mucus droplets. The right panel illustrates the network topology considering the possible locked configurations. Arrows entering a node indicate which nodes may block its oscillation. Arrows leaving a node indicate for which nodes its state, and mucus load is relevant to block the nodes the arrows are pointing at. The in-degree of a node is the sum of incoming arrows, and correspondingly, the out-degree is the sum of outgoing arrows. The grayscale in the graphs indicates the value of the in- and out-degrees and therewith the prevalent hierarchy among the nodes.

If we set open boundaries, marginal nodes in our networks are hardly influenced by their adjacent nodes. It is especially important that nodes at the left and right boundary have no in-degree considering the locked configurations rule. Consequently, these nodes would not adapt their oscillatory motions according to their neighbors’ motion and act as “leading nodes.”

Finally, we observed that some settings need a very regular topology with a prevalent hierarchy among the agents. If this “leadership” of a few nodes is abandoned, which happens when applying toric boundary conditions or importing unciliated cells, hardly any self-organization takes place.

#### Boundary-driven structure emergence hindered by unciliated cells

Unciliated cells can only take up and release mucus droplets and are thus, only passively involved in the state update of adjacent actuators. This means that cells adjacent to unciliated cells obtain a lower in-degree and therefore ascend the hierarchy of actuators. As we randomly distribute unciliated cells, “leading nodes” are no longer only found at the boundaries, but rather are distributed allover the network, and consequently, the network topology becomes less regular.

**Fig. 27:**
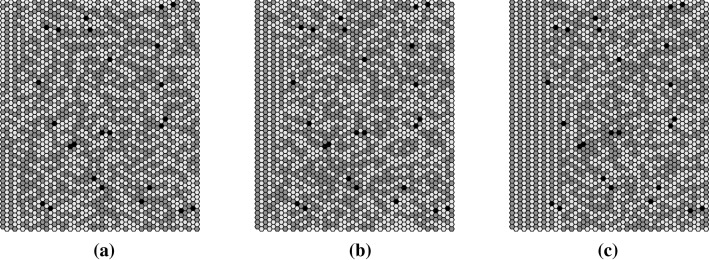
Three snapshots of the temporal evolution of the model when using the BHL + L alignment (time increases from **a**–**c**), open boundaries, RAU1 and 1% of randomly distributed unciliated cells. Note that the structure emergence is hindered by unciliated cells (which are black colored)

It has been observed that the introduction of unciliated cells hinders the spreading of the structure emergence for those settings for which the structure spreads strictly from an open boundary. Consequently, it seems that some settings require a very regular topology in order to be able to spread allover the network. The panels in Fig. 27 show three stages of a simulation for which we introduced 1$\%$ of unciliated cells and otherwise applied the same settings as in Fig. 25. Unciliated cells are shown in black. It is clearly visible that the structure emergence is hindered by the introduced unciliated cells, as the structure cannot spread further than to the first unciliated cells, seen from left. According to this simple observation, one could imagine that the influence of the boundaries gets the more restricted the more unciliated cells are incorporated. Further simulations have confirmed this idea.

#### Modular self-organization

**Fig. 28:**
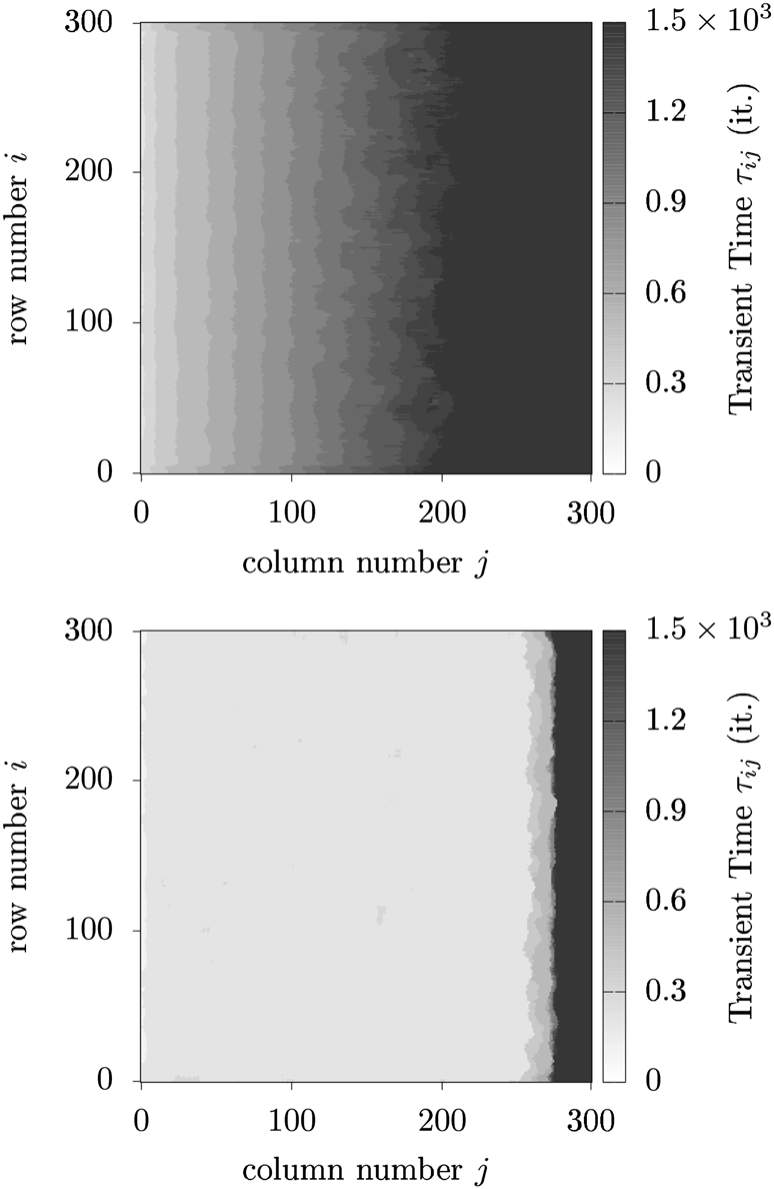
The grayscale indicates the locally resolved transient time [network updates] (note that there are two different grayscales). Both panels correspond to ensemble averages. The upper panel corresponds to runs having 100% ciliated cells, while the lower panel corresponds to runs with 90% of ciliated cells. The homogeneity in the lower panel indicates that boundary effects may be neglected in the network’s interior, if unciliated cells are considered

If we arrange the actuators in the square lattice (USL) and introduce a certain amount of unciliated cells, the array of actuators efficiently evolves to a self-cleaning epithelium. In this case, the network topology considering the state update gets strongly changed, what becomes obvious when thinking of the case of sparsely distributed ciliated cells. One can imagine that a group of ciliated cells would be surrounded by unciliated cells forming thereby “ciliated islands” on an otherwise unciliated epithelium. These islands would be hardly interconnected among each others when concerning the state updates. How strongly these modules are interconnected depends on the density of ciliated cells. In order to illustrate this modular character introduced by unciliated cells, the topology of the square lattice has been applied together with open boundary conditions and the update DAU for an ensemble with 100 members differing by their initial state. The grid size has been set to $300\times 300$ cells. Figure 28 illustrates the temporal evolution of the corresponding velocity fields. The grayscales illustrate the locally resolved transient time $\tau _{ij}$ (in effect the number of network updates it takes for an actuator to reach a local transport speed, which almost amounts to the final area-averaged transport speed). The upper panel in Fig. 28 corresponds to simulations ran with a dense mat of cells (100% ciliated cells), while in the lower panel 10% of randomly distributed unciliated cells were introduced. The panels represent the ensemble average of the spatially resolved transient times. The darker the color the longer it took until a certain actuator synchronized its movement. The discussed effect of the “leading boundary” is clearly visible in the upper panel of Fig. 28, as the gradient in brightness from the left to the right indicates the structure emergence, which sets in at the left boundary and spreads toward the right boundary. On the other hand, as soon as one introduces unciliated cells the self-organization process gets a modular character, as the structure emergence does not start at a certain point from which it spreads allover the network, but spreads at several locations simultaneously, which is illustrated by the relatively homogeneous distribution of grayscale in the lower panel in Fig. 28. The influence of the boundary is strongly restricted to the marginal nodes at both sides. The larger one chooses the grid size, or the more unciliated cells are introduced, the less important the “dominance” of the marginal actuators gets.

In conclusion, unciliated cells introduce a certain degree of modularity in the network topology as well as in the self-organizing process and therefore, the influence of the choice of boundary conditions can be neglected in the interior of the array, if the array size is chosen large enough and if a realistic amount of unciliated cells is considered.

The Online Resource 5 shows the evolution of the network’s state of a simulation run generated with ($100 \times 100$, USL, DAU, OP, 20%, 0.5, 10%) exhibiting the typical modular self-organization process leading to modular expression patterns.

#### Transient time versus network size

In this section, we point out that unciliated cells may strongly influence the dynamics of ciliated epithelia. As outlined in the previous section unciliated cells import topological modularity. Modularity is an important and promising concept, which is inter alia studied in terms of modular random Boolean networks. Poblanno-Balp and Gershenson (2011) claimed that topological modularity reduces the probability for damage spreading over the network, what promotes robustness.

In the following we show how unciliated cells affect the dynamics of our network model. As the square lattice is the only cell arrangement leading to a properly self-cleaning state, when introducing unciliated cells, all the results shown in this section refer to the square-lattice arrangement.

**Fig. 29:**
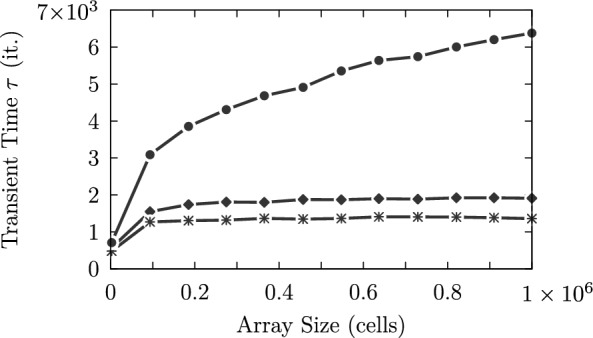
The graph shows the transient time [iterations] versus network size [cells]. The dots correspond to a totally dense ciliated mat. Diamonds and stars correspond to an array containing 98% and 90% ciliated cells, respectively

**Fig. 30:**
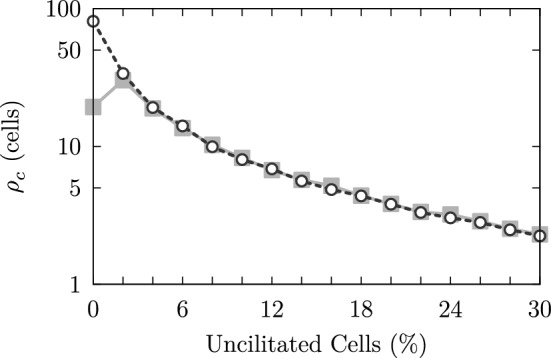
The graph shows the autocorrelation length [cells] versus the relative amount of unciliated cells [%] for different boundary conditions. The black circles and bright gray squares correspond to data points derived when using toric and open boundary conditions, respectively

The transient time has been determined for different array sizes in the range between $$50\times 50$$$$\hbox {cells}^2$$ and $$1000\times 1000$$$$\hbox {cells}^2$$ as well as for different fractions of unciliated cells (0%, 2% and 10%). The collected data points are presented in Fig. 29. The lines are only guidelines for the eye. The dots correspond to the dense mat case for which all cells are ciliated. Diamonds and stars correspond to 98% and 90% ciliated cells, respectively. One can clearly see that the transient time is not only reduced when introducing unciliated cells, but varies substantially different with increasing array size. While the transient time continuously grows for an array representing a totally ciliated mat with increasing array size, the transient time runs into saturation with increasing array size if unciliated cells are present. As discussed in the former section unciliated cells import not only topological modularity, but also cause the self-organization process to be modular, as the emergence of structure and transport evolves simultaneously in different modules. This finding most probably explains the level off of the transient time with increasing array size if unciliated cells are considered. As the self-organization process takes place in a decentralized manner, it does not depend upon the size of the actuator-array. On the other hand, the transient time continues to increase with increasing array size for an array purely consisting of ciliated cells, which is related to the nature of the structure emergence for these settings. According to the upper panel in Fig. 28 the self-organization process mainly starts at the left boundary, from where it spreads to the right allover the network. Accordingly, one would expect that the growth of the transient time continues with increasing network size, as it is the case in Fig. 29. (The transient time primarily depends on the length of the simulated actuator-array, as the ordered structures expand from the left to the right). As the transient time can be seen as a measure for robustness, our results suggest that the imported modularity promotes robustness. Consequently, it seems that the modular topology imports modularity into the self-organization process, which means that the organization of a huge network gets decomposed into a simultaneous organization of submodules. These submodules can be recognized by examining the spatial structure of the network’s state, as it has been observed that the modularity of the expression patterns clearly depend on the density of ciliated cells. The autocorrelation length of attracting network states has been determined for settings differing by the amount of ciliated cells as well as the boundary conditions. As we are interested in the spatial structures of the expression patterns, the grid size has been chosen to $$200 \times 200$$ cells. Figure 30 shows the autocorrelation length $$\rho _c$$ (cells) versus the relative amount of unciliated cells (%) for open and toric boundary conditions. It can be clearly seen that the autocorrelation length decreases with an increasing portion of unciliated cells. Furthermore, the autocorrelation length is roughly given by the mean distance of unciliated cells. Consequently, unciliated cells may play a further role in the self-organization process on the ciliated airway epithelium, as the appearance of the previously described patch-work character may be strongly influenced by the distribution of unciliated and ciliated cells.

## Discussion

The aim of this study is twofold: on the one hand, we want to make the self-organized spatiotemporally coordinated ciliary beat patterns as well as the self-organized fluid transport across multiciliated epithelia plausible. We suggest that the cooperation among ciliated cells emerges from locally interacting oscillating cilia bundles belonging to different ciliated cells. As our goal was to keep our model as simple as possible, we present a virtual self-cleaning epithelium model based on symmetrically interacting two-state actuators, which we formulate in terms of an adaptive Boolean network. In the framework of adaptive Boolean networks, the oscillatory motion of ciliated cells can be represented by “blinking nodes” and discrete mucus droplets establish the local interactions and therefore the network’s topology. In “Coevolution of spatiotemporal patterns and transport” section, we demonstrate the coevolution of the network’s state and its topology, which is a characteristic property of adaptivity and represents the self-organized coevolution of ciliary beating patterns and associated fluid transport.

On the other hand, we report our insights to our system’s dynamics we gained by conducting parameter studies. In the following, we discuss the observed effects of the update scheme, the boundary conditions and the amount of unciliated cells on the dynamics of our network model.

As we formulate our epithelium model in terms of a discretized asynchronous multi-agent network, the question of how to update the network arises. The introduced update schemes are meant to represent different possible intercellular signaling mechanisms (membrane potentials and calcium waves). Only settings using either the deterministic asynchronous update (DAU) or the alignment BHL + L, or both, guide the network toward properly self-cleaning states (Table 1). Settings using BHL + L or DAU introduce a local recurrent temporal coupling among adjacent actuators inducing an asymmetry of the local interactions. Figure 19 suggests that the prevalent asymmetry, such as the initial speed associated with a specific parameter setting, increases the probability for efficient self-organization toward self-cleaning states. In any case none of the roughly 11,000 simulations exhibiting an initial transport speed of less than 0.01 cells/it self-organizes efficiently toward a properly self-cleaning state. The fundamental role of asymmetric interactions on extended systems has been discussed previously. Asymmetry-induced effects on the synchronization process of a pair of coupled fields have been reported by Boccaletti et al. (2005), where it has been particularly argued that small changes in the asymmetry of the interactions could be used as an efficient way to synchronize or desynchronize the dynamics, as well as select the main statistical properties of the synchronized motion in ensembles of interacting units and consequently, may have relevant consequences in natural systems. The synchronization process of a ciliary chain attached to a cylindrical surface has been investigated by Ghorbani and Najafi (2017). Each cilium is modeled in terms of a small sphere moving along an elliptic trajectory. It has been shown that an asymmetry in their orbits triggers the emergence of metachronal waves. Symmetrical settings have not shown any correlations in their beating patterns, what compares well to our results.

Furthermore, the application of the deterministic update scheme (DAU) seems to generate less well-ordered attracting network states. This behavior is exemplary illustrated in Fig. 23 showing a perfectly ordered attracting state for the random asynchronous update (RAU1), slightly less well-ordered network states for the semi-random update schemes (SRAU1 and SRAU2) and finally, the least ordered attracting states for the deterministic update scheme (DAU). All settings using the deterministic update scheme have consistently generated patterns showing “defects” perpendicular to the direction of the update scheme and maximum correlation along the direction of the update. Figure 15 represents an autocorrelogram of an extreme case: maximum correlation is found into the direction of the primary update direction (from right to left), which is most probably caused by the local temporal coupling among actuators, while there seems to be almost no local temporal coupling from the top to the bottom, what leads to typically elongated autocorrelograms if DAU is applied.

Finally, the less strict organization the network generates when using DAU may lead to more flexible dynamics as in the case of a perturbation the system does not simply recover its original attracting state, but conforms to the changes by running into a completely different attractor (see Fig. 24).

The effects of different update schemes on the dynamics of multi-agent systems are still being investigated. Cornforth et al. (2005) applied six different update schemes on one-dimensional cyclic cellular automata to compare the resulting dynamics. It has been concluded that deterministic update schemes confer a degree of flexibility upon the system dynamics, what compares well to the observed conforming character of our model settings using a deterministic update scheme. Consequently, so far, the findings show evidence that in various asynchronous processes leading to self-organization, a deterministic update scheme leads to more realistic dynamics (flexibility and robustness) and may therefore be favored by evolution (Gershenson 2004b). Recall, however, that “deterministic” does not mean that actuators would displace the mucus in a pre-determined direction. Rather, the transport direction evolves through the interplay between the update asymmetry and the largely stochastic interactions.

Finally, we would like to point out the possible effect of unciliated cells on the dynamics on ciliated epithelia. The topology of our network model is primarily given by the formulation of the local interaction rules, the choice of the boundary conditions and the amount of unciliated cells. As we have outlined in “Modular self-organization” section the amount of unciliated cells introduces a certain degree of topological modularity. The topological modularity in turn causes a modular self-organization, which means that the self-organization does not start at a specific point or boundary on the grid—as it has been observed for completely dense mats of ciliated cells—but starts in each module simultaneously. This modular character of the self-organization leads to the size independence of the transient time, reported in “Transient time versus network size” section. Consequently, modularity may provide robustness even to networks as large as the human ciliated airway epithelium consisting of more than $10^9$ cells (Mercer et al. 1994), as perturbations quickly fade away. Furthermore, the modular topology leads to modular expression patterns, the size of which is roughly given by the mean distance of unciliated cells (as shown in Fig. 30). Finally, the finding of a modular self-organization caused by the underlying modular topology providing a highly robust patch-work among actuators provides a consistent explanation of the modular expression patterns previously reported in experimental studies aimed at the quantitative description of the modulation wave fields on the tracheal epithelium (Ryser et al. 2007).

As very recently pointed out by Dey et al. (2018), theoretical studies investigating the collective dynamics of hydrodynamically interacting cilia have, so far, usually considered homogeneous carpets of cilia. Dey et al. (2018) investigated the role of a spatial heterogeneous ciliary distribution on coherent ciliary beating using one-dimensional arrays of cilia represented by rowers. It is particularly shown that the phase coherence of random clustered distributions of rowers is less sensitive to variations of the number density than (homogeneously) random distributions of rowers. This finding might be seen as another specific dynamical phenomenon improving robustness by an underlying modular (network) topology.

We conclude that an intercellular signaling mechanism is probable on ciliated epithelia, as deterministic update schemes drive the model toward robust self-organized states, which can still conform to changes. We suggest that the patchy expression patterns of the modulation wave field observed on real ciliated epithelia may be the result of the underlying modular topology, which is primarily formed by the distribution of ciliated and unciliated cells. This patch-work character among ciliated cells may be highly robust due to a modular self-organization, which prevents perturbations to spread over the whole network. Furthermore, the boundary conditions may become irrelevant on epithelia being either large enough or having a low amount of ciliated cells.

We close this study by hypothesizing that the modular organization of the dynamics on ciliated epithelia may be seen as a robust size-independent construction plan of nature, which leads to properly self-cleaning airways in organisms being as small as new born mice as well as in adult giraffes.

## Electronic supplementary material

Below is the link to the electronic supplementary material.

Supplementary material 1 (mpeg 14116 KB)

Supplementary material 2 (mpeg 11281 KB)

Supplementary material 3 (mp4 14893 KB)

Supplementary material 4 (mp4 171520 KB)

Supplementary material 5 (mpeg 5852 KB)

## Footnotes

1 Oscillations of 0.5 in the normalized Hamming distance (caused by the XOR function and the BHL + L setting) are suppressed by taking the minimum of four consecutive Hamming distances.

## References

[CR1] Aldana M, Coppersmith S, Kadanoff LP (2003) Boolean dynamics with random couplings. In: Perspectives and problems in nonlinear science. Springer, New York, pp 23–89. 10.1016/S0167-2789(03)00174-X

[CR2] Boccaletti S, Mendoza C, Bragard J (2005). Synchronization of spatially extended chaotic systems with asymmetric coupling. Braz J Phys.

[CR3] Burgess SA, Walker ML, Sakakibara H, Knight PJ, Oiwa K (2003). Dynein structure and power stroke. Nature.

[CR4] Cornforth D, Green DG, Newth D (2005). Ordered asynchronous processes in multi-agent systems. Physica D.

[CR5] De Iongh R, Rutland J (1989). Orientation of respiratory tract cilia in patients with primary ciliary dyskinesia, bronchiectasis, and in normal subjects. J Clin Pathol.

[CR6] Dey S, Massiera G, Pitard E (2018). Role of spatial heterogeneity on the collective dynamics of cilia beating in a minimal 1D model. Phys Rev E.

[CR7] Elgeti J, Gompper G (2013). Emergence of metachronal waves in cilia arrays. Proc Nat Acad Sci USA.

[CR8] Geiser M, Im Hof V, Siegenthaler W, Grunder R, Gehr P (1997). Ultrastructure of the aqueous lining layer in hamster airways: is there a two-phase system?. Microsc Res Tech.

[CR9] Gershenson C (2002) Classification of random boolean networks. In: Standish RK, Bedau MA, Abbass HA (eds) Artificial Live VIII: Proceedings of the eight international conference on artificial life. MIT Press, pp 1–8

[CR10] Gershenson C (2004a) Introduction to random boolean networks. In: Bedau M, Husbands P, Hutton T, Kumar S, Suzuki H (eds) Workshop and Tutorial Proceedings, ninth international conference on the simulation and synthesis of living systems (ALife IX), pp 160–173. https://arxiv.org/abs/nlin/0408006

[CR11] Gershenson C (2004b) Updating schemes in random boolean networks: do they really matter? In: Pollack J, Bedau M, Husbands P, Ikegami T, Watson R (eds) Artificial Life IX, Proceedings of the ninth international conference on the simulation and synthesis of living systems. MIT Press, pp 238–243. http://uk.arxiv.org/abs/nlin.AO/0402006, arXiv:0402006v3

[CR12] Ghorbani A, Najafi A (2017). Symplectic and antiplectic waves in an array of beating cilia attached to a closed body. Phys Rev E.

[CR13] Greil F (2009) Dynamics of boolean networks. Ph.D. thesis, T.U. Darmstadt. 10.3934/dcdss.2011.4.1629. arXiv:1307.0757

[CR14] Greil F (2012). Boolean networks as modeling framework. Front Plant Sci.

[CR15] Gross T, Blasius B (2008). Adaptive coevolutionary networks: a review. J R Soc Interface.

[CR16] Gueron S, Levit-Gurevich K, Liron N, Blum JJ (1997). Cilia internal mechanism and metachronal coordination as the result of hydrodynamical coupling. Proc Nat Acad Sci USA.

[CR17] Hanel R, Thurner S, Gell-Mann M (2014). How multiplicity determines entropy and the derivation of the maximum entropy principle for complex systems. Proc Natl Acad Sci.

[CR18] Hilfinger A, Jülicher F (2008). The chirality of ciliary beats. Phys Biol.

[CR19] Hilfinger A, Chattopadhyay AK, Jülicher F (2009). Nonlinear dynamics of cilia and flagella. Phys Rev E Stat Nonlinear Soft Matter Phys.

[CR20] Lee CH, Lee SS, Mo JH, Kim IS, Quan SH, Wang SY, Yi WJ, Rhee CS, Min YG (2005). Comparison of ciliary wave disorders measured by image analysis and electron microscopy. Acta Oto-Laryngol.

[CR21] Lee W, Jayathilake P, Tan Z, Le D, Lee H, Khoo B (2011). Muco-ciliary transport: effect of mucus viscosity, cilia beat frequency and cilia density. Comput Fluids.

[CR22] Linck RW (2009). Cilia and flagella. Encyclopedia of life sciences.

[CR23] Marshall WF (2010). Cilia self-organize in response to planar cell polarity and flow. Nat Cell Biol.

[CR24] Mercer RR, Russell ML, Roggli VL, Crapo JD (1994). Cell number and distribution in human and rat airways. Am J Respir Cell Mol Biol.

[CR25] Mitran SM (2007). Metachronal wave formation in a model of pulmonary cilia. Comput Struct.

[CR26] Niedermayer T, Eckhardt B, Lenz P (2008). Synchronization, phase locking, and metachronal wave formation in ciliary chains. Chaos.

[CR27] Oliveira MJR (2003). Zonation of ciliated cells on the epithelium of the rat trachea. Lung.

[CR28] Plopper CG, Mariassy AT, Wilson DW, Alley JL, Nishio SJ, Nettesheim P (1983). Comparison of nonciliated tracheal epithelial cells in six mammalian species: ultrastructure and population densities. Exp Lung Res.

[CR29] Poblanno-Balp R, Gershenson C (2011). Modular random Boolean networks. Artif Life.

[CR30] Purcell EM (1977). Life at low Reynolds number. Am J Phys.

[CR31] Rautiainen MEP (1988). Orientation of human respiratory cilia. Eur Respir J.

[CR32] Ricka J (2010). Zilien, von Molekularmotoren zur selbstorganisierten Selbstreinigung der Atmungswege. Physik in unserer Zeit.

[CR33] Riedel-Kruse IH, Hilfinger A, Howard J, Jülicher F (2007). How molecular motors shape the flagellar beat. HFSP J.

[CR34] Rohlf T, Bornholdt S (2009) Self-organized criticality and adaptation in discrete dynamical networks. In: Adaptive networks. Springer, Berlin, pp 73–106, http://uk.arxiv.org/abs/0811.0980v1, arXiv:0811.0980v1

[CR35] Ross SM, Corrsin S (1974). Results of an analytical model of mucociliary pumping. J Appl Physiol.

[CR36] Rutland J, De Iongh RU (1993). Random ciliary orientation. N Engl J Med.

[CR37] Ryser M, Burn A, Wessel T, Frenz M, Rička J (2007). Functional imaging of mucociliary phenomena: high-speed digital reflection contrast microscopy. Eur Biophys J.

[CR38] Salathe M (2007). Regulation of mammalian ciliary beating. Annu Rev Physiol.

[CR39] Satir P, Christensen ST (2007). Overview of structure and function of mammalian cilia. Annu Rev Physiol.

[CR40] Smith DJ, Ea G, Blake JR (2007). A viscoelastic traction layer model of muco-ciliary transport. Bull Math Biol.

[CR41] Smith DJ, Ea G, Blake JR (2007). Discrete cilia modelling with singularity distributions: application to the embryonic node and the airway surface liquid. Bull Math Biol.

[CR42] Sui H, Downing KH (2006). Molecular architecture of axonemal microtubule doublets revealed by cryo-electron tomography. Nature.

[CR43] Van As A, Webster I (1974). The morphology of mucus in mammalian pulmonary airways. Environ Res.

[CR44] Wong LB, Miller IF, Yeates DB (1993). Nature of the mammalian ciliary metachronal wave. J Appl Physiol.

[CR45] Wuensche A (1998). Discrete dynamical networks and their attractor basins. Complex Int.

[CR46] Yi WJ, Park KS, Lee CH, Rhee CS, Nam SW (2002). Directional disorder of ciliary metachronal waves using two-dimensional correlation map. IEEE Trans Bio-med Eng.

